# Occupational and domestic exposure associations with cerebral small vessel disease and vascular dementia: A systematic review and meta‐analysis

**DOI:** 10.1002/alz.13647

**Published:** 2024-01-25

**Authors:** Una Clancy, Yajun Cheng, Amrita Brara, Fergus N. Doubal, Joanna M. Wardlaw

**Affiliations:** ^1^ Centre for Clinical Brain Sciences and the UK Dementia Research Institute Chancellor's Building University of Edinburgh Edinburgh UK; ^2^ Center of Cerebrovascular Diseases Department of Neurology West China Hospital Sichuan University Chengdu Sichuan China

**Keywords:** cerebral small vessel disease, dementia, occupations, public health, stroke, white matter hyperintensities, workplace

## Abstract

**INTRODUCTION:**

The prevalence of cerebral smallvessel disease (SVD) and vascular dementia according to workplace or domestic exposure to hazardous substances is unclear.

**METHODS:**

We included studies assessing occupational and domestic hazards/at‐risk occupations and SVD features. We pooled prevalence estimates using random‐effects models where possible, or presented a narrative synthesis.

**RESULTS:**

We included 85 studies (*n* = 47,743, mean age = 44·5 years). 52/85 reported poolable estimates. SVD prevalence in populations exposed to carbon monoxide was 81%(95% CI = 60‐93%; *n* = 1373; results unchanged in meta‐regression), carbon disulfide73% (95% CI = 54‐87%; *n* = 131), 1,2‐dichloroethane 88% (95% CI = 4‐100%, *n* = 40), toluene 82% (95% CI = 3‐100%, *n* = 64), high altitude 49% (95% CI = 38‐60%; *n* = 164),and diving 24% (95% CI = 5‐67%, *n* = 172). We narratively reviewed vascular dementia studies and contact sport, lead, military, pesticide, and solvent exposures as estimates were too few/varied to pool.

**DISCUSSION:**

SVD and vascular dementia may be associated with occupational/domestic exposure to hazardous substances. CRD42021297800.

## 1 INTRODUCTION

Cerebral small vessel disease (SVD) is a common cause of dementia and stroke,^1^ but its etiology is poorly understood. Historically, researchers have focused on vascular risk factor contributions to SVD but these only explain 2% of the variance in white matter hyperintensities (WMH),^2^ a key feature of SVD. We need to consider alternative risks.

Early life factors, including education, intelligence, and childhood socioeconomic status, are linked with brain vascular disease, including lifetime stroke and dementia risk^3^, ^4^ and severity of cerebrovascular disease on neuroimaging.^5^ However, midlife risk factors need closer scrutiny. Occupation is a prolonged lifetime exposure, yet associations between occupation, SVD, dementia, and stroke are understudied. Moreover, we spend most of our lives in our homes, but domestic exposures to hazardous substances have not been studied. No published systematic review or meta‐analysis has assessed relationships between occupational/domestic exposures and SVD or vascular dementia (VaD).

Occupational and domestic exposures could be midlife contributors to SVD and VaD. We aimed to determine whether individuals who work in high‐risk occupations or who experience workplace or domestic exposure to hazardous substances have (a) high prevalence of SVD or VaD and (b) higher risk of SVD or VaD versus unexposed populations.

## 2 METHODS

### 2.1 Search strategy and selection criteria

We searched MEDLINE and EMBASE from inception to March 1, 2023, for studies investigating associations between a broad range of potentially hazardous occupational exposures or settings and SVD or VaD (eText 1). We included studies reporting SVD or VaD prevalence or risk in occupationally exposed populations. We followed Preferred Reporting in Systematic Reviews and Meta–Analysis guidance (eText 2). We hand‐searched reference lists of included articles.

We searched broadly to detect individuals exposed to hazardous workplace substances or settings, based on occupational hazards identified by the UK Health and Safety Executive, Control of Substances Hazardous to Health Regulations, and EU Occupational Safety and Health Agency.^6^, ^7^, ^8^ See Supplementary Text 1 for the search strategy which includes exposure to specific substances, for example, carbon monoxide, diesel; activities, for example, coal‐mining, welding; and settings, for example, agriculture, shipyards.

We included domestic exposures and substance misuse of hazardous substances found in workplaces to capture high‐concentration exposures, for example, carbon monoxide, toluene inhalation.

We included peer‐reviewed full‐text studies assessing SVD according to the presence versus absence of clinical, radiological, or pathological SVD features in adults (>18 years). We defined clinical features as lacunar stroke or VaD. We defined radiological features on MRI or CT according to the STandards for ReportIng Vascular changes on nEuroimaging (STRIVE), that is, WMH, microbleeds, perivascular spaces (PVS), lacunes, lacunar infarcts and small subcortical infarcts.^9^ We included *post mortem* studies describing pathological SVD features.^10^ We did not restrict study design or language. We excluded genetic SVD and other neurodegenerative conditions (for example, multiple sclerosis) and case series <5.

Two reviewers (U.C., Y.C.) independently screened titles, abstracts, reviewed full texts, and extracted data, using Covidence.^11^ We resolved disagreements through consensus with wider team discussion if necessary (J.M.W., F.N.D.). We did not contact authors about missing data. We used RoBANS (risk of bias assessment tool for non‐randomized studies) tool to assess study quality.^12^ We registered the protocol (PROSPERO CRD: 42021297800; 14^th^ December 2021).

### 2.2 Data analysis

#### 2.2.1 Data extraction

Two reviewers (U.C., Y.C.) extracted population, occupational or substance exposure, exposure setting, study design, sample size, mean age (years), imaging modality, SVD features/terminology/prevalence, summary estimates, exposure duration/concentration, exposure‐imaging interval, blinding, and adjustment for age, socioeconomics, and vascular risk factors. In duplicate populations, we selected the larger sample size assessing SVD.

We extracted the following summary estimates, using the highest‐adjusted estimates available, and grouped analyses according to exposure category: (1) raw data on (a) total population SVD prevalence in observational studies where the full study population was occupationally exposed and (b) SVD prevalence in case‐control studies according to exposed versus unexposed groups; (2) SVD rating or volume, including mean differences where available; (3) Odds ratios (ORs) comparing SVD presence versus absence, or VaD presence versus absence. We extracted each radiological SVD feature, for example, WMH, PVS, microbleeds, and clinical feature, that is, VaD or lacunar stroke, where available.

**RESEARCH IN CONTEXT**

1. **Systematic review**: Occupational and domestic exposures have not been explored yet as a potential midlife risk factor for cerebral small vessel disease (SVD), vascular dementia, or small vessel strokes. We did not find any systematic reviews or meta‐analyses assessing associations between occupation or domestic exposures and SVD.
2. **Interpretation**: This systematic review and meta‐analysis of 85 studies assessing > 11 occupational exposures (47,743 participants) shows that certain occupational and domestic exposure to hazardous substances and settings may be associated with SVD and vascular dementia.
3. **Future directions**: Brain health implications of occupational exposures may be being overlooked currently. Our findings of novel substance/setting associations with SVD and vascular dementia can be used to drive occupational health, dementia and stroke research into threshold safety limits and outcomes according to specific workplace exposures and preventative measures. We need epidemiological studies assessing dose‐response relationships across the life‐course.

#### 2.2.2 Meta‐analysis

For meta‐analysis, we extracted SVD prevalence data and pooled prevalence estimates for each occupational category. After extracting raw frequencies, we logit‐transformed and pooled proportions using generalized linear mixed‐effects models. We used Knapp–Hartnung adjustments to calculate 95% confidence intervals (CI) of pooled effects.^13^ We used random‐effects models as we anticipated heterogeneity. Due to limited case‐control study numbers per category, we were unable to pool case‐control studies separately, so we included exposed cases only in our meta‐analyses to enable pooling of prevalence data with observational studies of entire exposed cohorts. However, we also narratively synthesized case‐control studies separately, comparing exposed, versus unexposed groups. We report findings as pooled SVD prevalence (%) with 95% CI using forest plots. We performed meta‐regression to investigate age effects where >10 studies were available. We defined heterogeneity using *I* ^2^: low (25%), moderate (50%), or high (75%). We performed quantitative analyses using R package meta.^14^ For meta‐analyses, we extracted cross‐sectional baseline data. We narratively synthesized longitudinal findings.

#### 2.2.3 Narrative synthesis

We reported studies not meta‐analyzed in narrative synthesis. We had planned to extract and meta‐analyze OR but were unable due to infrequent OR reporting by category of occupational exposure. Instead, we report OR in narrative synthesis.

#### 2.2.4 Risk of bias assessment

We used RoBANS (risk of bias assessment tool for non‐randomized studies)^12^ to assess risk of bias, rated as high/low/unclear based on: participant selection; adjustment for confounders; exposure measurement adequate; blinding of outcome assessment; incomplete outcome data; and selective outcome reporting. U.C. assessed study quality and Y.C. independently assessed a random 33% sample.

#### 2.2.5 Role of the funding source

The study funders had no role in design, data collection, analysis, interpretation, or writing of the report. The corresponding author had access to study data and had final responsibility in deciding to submit for publication.

#### 2.2.6 Data availability

Anonymized data not published within this article can be made available by request from any qualified investigator.

## 3 RESULTS

### 3.1 Characteristics of included studies and populations

**FIGURE 1:**
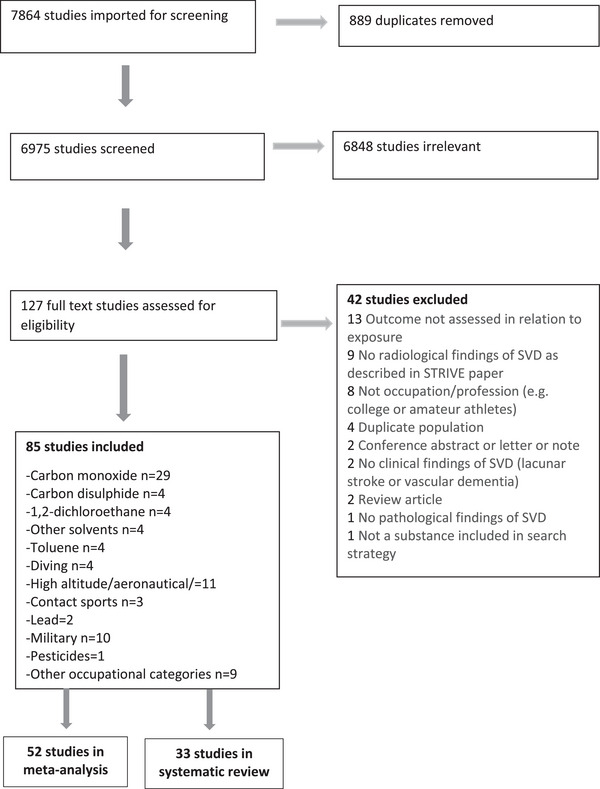
The Preferred Reporting Items for Systematic Reviews and Meta‐Analyses (PRISMA) flow diagram of study selection.

After removing 889 duplicates, our search identified 6,975 papers. Following abstract screening, 127/6,975 were eligible for full‐text review. We excluded 42/127 full‐texts, primarily because SVD was not assessed in relation to occupational exposure or SVD was not described. We identified 85 papers describing 85 populations for inclusion, with total 47,743 participants (1,944 in meta‐analyses), of mean age = 44·5 years. Mean age was unreported for two studies. Seventy studies were observational and 15 were case‐control. See Figure 1 for Preferred Reporting Items for Systematic Reviews and Meta‐Analyses (PRISMA) diagram and Supplementary Table 1 for summary of included studies.

Exposures included carbon monoxide (29 studies, *n* = 1,409; 28 studies/*n* = 1,373 in meta‐analysis),^15^, ^16^, ^17^, ^18^, ^19^, ^20^, ^21^, ^22^, ^23^, ^24^, ^25^, ^26^, ^27^, ^28^, ^29^, ^30^, ^31^, ^32^, ^33^, ^34^, ^35^, ^36^, ^37^, ^38^, ^39^, ^40^, ^41^, ^42^, ^43^ carbon disulfide (4 studies, *n* = 797; 3 studies/*n* = 131 in meta‐analysis),^44^, ^45^, ^46^, ^47^ 1,2‐dichloroethane (4 studies, *n* = 40, *n* = 40 in meta‐analysis),^48^, ^49^, ^50^, ^51^ toluene (4 studies, *n* = 64/*n* = 64 in meta‐analysis),^52^, ^53^, ^54^, ^55^ diving (4 studies, *n* = 266; 4 studies/*n* = 172 in meta‐analysis),^56^, ^57^, ^58^, ^59^ high altitude (11 studies, *n* = 950; 5 studies/*n* = 164 in meta‐analysis),^60^, ^61^, ^62^, ^63^, ^64^, ^65^, ^66^, ^67^, ^68^, ^69^, ^70^ contact sports (3 studies, *n* = 629; not meta‐analyzable),^71^, ^72^, ^73^ lead (2 studies, *n* = 423, not meta‐analyzable),^74^, ^75^ military (10 studies, *n* = 18,893, not meta‐analyzable),^76^, ^77^, ^78^, ^79^, ^80^, ^81^, ^82^, ^83^, ^84^, ^85^ pesticides/fertilizers (1 study, *n* = 8623, not meta‐analyzable),^86^ miscellaneous solvents (4 studies, *n* = 251, not meta‐analyzable),^87^, ^88^, ^89^, ^90^ and broad occupational categories in population‐based dementia studies (9 studies, *n* = 15,118, not meta‐analyzable).^91^, ^92^, ^93^, ^94^, ^95^, ^96^, ^97^, ^98^, ^99^

Imaging‐exposure interval was ongoing/recent (same day‐3 months) in 46, past (≥3 months‐years) in 25, and unspecified in 14 studies; range same day to 33 years; not reported in 31/85.

Fifty‐one exposures were occupational, 15 were domestic including accidental/deliberate, and 19 studies, all involving carbon monoxide poisoning, did not specify occupational versus domestic exposure.

SVD imaging features were reported in 71 studies, VaD diagnosis in 12 studies,^91^, ^92^, ^93^, ^94^, ^95^, ^96^, ^97^, ^98^, ^99^ and *post mortem* SVD in 2 studies.^59^, ^88^ VaD subtypes (e.g., subcortical) were not reported. No study assessed lacunar stroke syndromes. Of 71 imaging studies, 56 had MRI, 11 CT/MRI, and 4 CT. Of 85 studies, 66 were cross‐sectional, 9 had longitudinal imaging, and 10 were mainly cross‐sectional with a longitudinal subset. Longitudinal data were available for 10 carbon monoxide,^16^, ^18^, ^21^, ^23^, ^25^, ^26^, ^32^, ^35^, ^37^, ^41^ 1 carbon disulfide,^46^ 4 high altitude,^62^, ^66^, ^69^, ^70^ 1 lead,^75^ 2 military,^78^, ^84^ and 1 toluene study.^52^

See Supplementary Table 2 for terms used to describe SVD features. Of studies reporting radiological SVD features, 59/71 described SVD presence versus absence. Of 12/71 remaining, 3 reported WMH volumes, 3 reported PVS volume change, 2 reported WMH volume change, 1 reported WMH number, 1 reported both WMH volume/number, 1 reported diffuse foci within periventricular white matter and centrum semioval, and 1 reported WMH Cardiovascular Health Score change.

### 3.2 Risk of bias

We outline risk of bias assessment in Supplementary Table 3. Six studies had low risk of bias across all domains. The main biases were non‐adjustment for confounders, for example, age and reporting on image‐rater blinding to occupational exposures.

### 3.3 Meta‐analyses

Most studies reported descriptive statistics, for example, unadjusted frequencies. Due to insufficient study numbers and/or heterogeneous reporting of occupations and/or hazardous substances, it was only possible to meta‐analyze SVD prevalence, not severity/non‐WMH features. See in Supplementary Table 4 for number of meta‐analyzed, versus, narratively synthesized studies.

#### 3.3.1 Carbon monoxide

**FIGURE 2:**
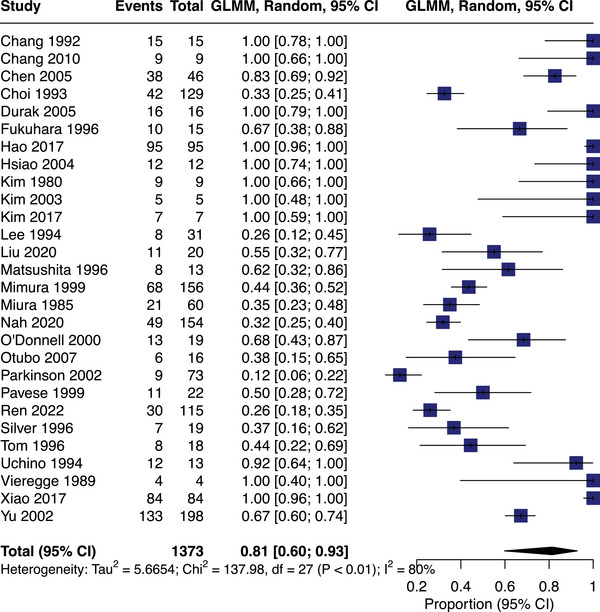
Meta‐analysis of small vessel disease prevalence in studies assessing exposure to carbon monoxide (in alphabetical author order with year of study publication).

Twenty‐nine studies assessed carbon monoxide associations with SVD (*n* = 1,409, with meta‐analyzable data for *n* = 1,373 from 28 studies). The pooled SVD prevalence in carbon monoxide‐exposed populations was 81% (95% CI = 60‐93%; mean age = 45; Figure 2).

One carbon monoxide study did not provide SVD prevalence data^39^ but described diffuse hypodensity in cerebral white matter (CT) and hyperintense periventricular and centrum semioval foci (MRI) in encephalopathic patients following carbon monoxide poisoning (*n* = 20, mean age = 41·5). Nine exposures were domestic,^17^, ^18^, ^24^, ^25^, ^26^, ^27^, ^32^, ^36^, ^40^ one occupational,^31^ and, in 19 studies, all acute, exposure settings were unclear.

#### 3.3.2 Carbon disulfide

**FIGURE 3:**
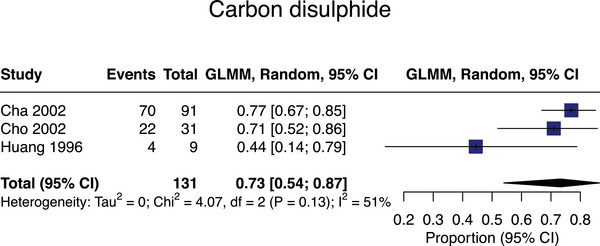
Meta‐analysis of small vessel disease prevalence in studies assessing exposure to carbon disulfide.

Four studies assessed carbon disulfide associations with SVD,^44^, ^45^, ^46^, ^47^ *n* = 797, with meta‐analyzable data for *n* = 131 from three studies.^44^, ^45^, ^47^ The pooled SVD prevalence in carbon disulfide‐exposed populations was 73% (95% CI = 54‐87%; mean age = 51, Figure 3).

One case‐control study not meta‐analyzed due to unreported prevalence data^46^ in rayon factory workers in Japan (324 exposed to carbon disulfide and 342 unexposed but manufacturing other synthetic fibers/plastics) showed that 6‐year increase in number of T2‐weighted “hyperintense spots” was higher in workers with ongoing (OR = 2·27 [95% CI = 1·37‐3·76]) versus previous exposure (OR = 1·33 [95% CI = 0·70‐2·54]) versus unexposed workers. All carbon disulfide exposures occurred in an occupational setting.

#### 3.3.3 1,2‐dichloroethane

**FIGURE 4:**
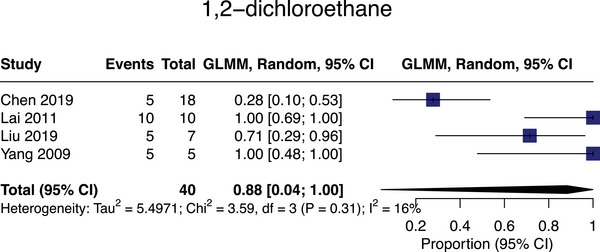
Meta‐analysis of small vessel disease prevalence in studies assessing exposure to 1,2‐dichloroethane.

Four studies assessed 1,2‐dichloroethane associations with SVD,^48^, ^49^, ^50^, ^51^ *n* = 40, with meta‐analyzable data for all. The pooled SVD prevalence in 1,2‐dichloroethane‐exposed populations was 88% (95% CI = 4‐100%; mean age = 34; Figure 4). All 1,2‐dichloroethane exposures were occupational.

#### 3.3.4 Toluene

**FIGURE 5:**
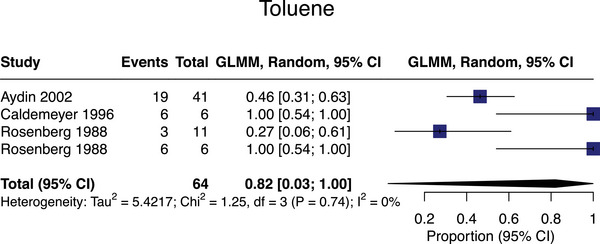
Meta‐analysis of small vessel disease prevalence in studies assessing exposure to toluene.

Four studies assessed toluene associations with SVD,^52^, ^53^, ^54^, ^55^ *n* = 64, all meta‐analyzable. The pooled SVD prevalence in toluene‐exposed populations was 82% (95% CI = 3%‐100%; mean age = 28; Figure 5). All toluene exposures were in a non‐occupational intentional abuse setting.

#### 3.3.5 Sub‐aqua diving

**FIGURE 6:**
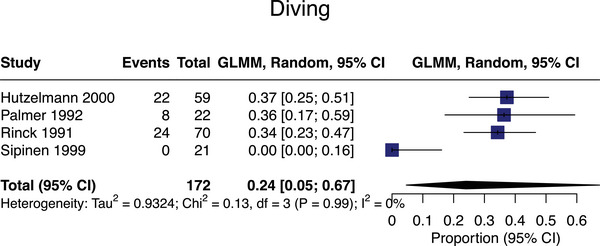
Meta‐analysis of small vessel disease prevalence in studies assessing exposure to diving.

Four studies assessed diving associations with SVD,^56^, ^57^, ^58^, ^59^ *n* = 266, with meta‐analyzable data for *n* = 172 exposed cases from four studies. The pooled SVD prevalence in diving‐exposed populations was 24% (95% CI = 5%‐67%; mean age = 38; Figure 6). All diving exposures were occupational.

#### 3.3.6 High altitude

**FIGURE 7:**
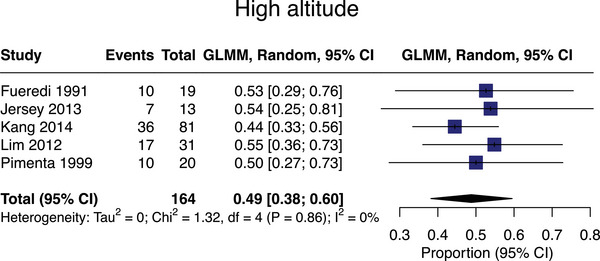
Meta‐analysis of small vessel disease prevalence in studies assessing exposure to high altitude.

Eleven studies assessed high‐altitude associations with SVD,^60^, ^61^, ^62^, ^63^, ^64^, ^65^, ^66^, ^67^, ^68^, ^69^, ^70^ *n* = 950, with meta‐analyzable data for *n* = 164 exposed cases from 5 studies.^60^, ^63^, ^64^, ^65^, ^67^ The pooled SVD prevalence in high altitude‐exposed populations was 49% (95% CI = 38%‐60%; mean age = 42; Figure 7).

Six studies were not meta‐analyzable^61^, ^62^, ^66^, ^68^, ^69^, ^70^ due to limited ability to pool extractable estimates. A case‐control study (*n* = 336, mean age = 37)^68^ found altitude personnel (*n* = 83) and high‐altitude pilots (*n* = 105) had greater volumes/numbers of WMH (mean volume 0·12 mL [SD = 0·4 mL], *p* = 0·01); mean count = 6·4 [SD = 11·1], *p* = 0·01; pilots’ mean volume = 0·14 mL [SD 0·29], *p* < 0·001); mean number = 9·7 [SD = 18·3], *p* < 0·001) versus 148 healthy controls (mean volume = 0·03 mL [SD = 0·05]; mean count = 2·6 [SD = 3·1]). Another case‐control study ^62^ (*n* = 124, mean age = 21) performed MRI on 64 airforce crew at 24 and 72 hours after altitude training. There were no WMH changes but significant increases in white matter blood flow were detected 24 hours post‐exposure. A case‐control study ^61^ (*n* = 193, mean age = 37) comparing 102 high‐altitude pilots with hypobaric exposure versus 91 active military controls found pilots had higher WMH volume (0·15 mL [SD = 0·3] vs. 0·04 mL [SD = 0·07], *p* = 0·004) and number (9·78 [SD = 18·2] vs. 3·29 [SD = 4·49], *p* < 0·001) versus controls. Cases and controls in all were age‐matched but analyses were unadjusted.

A longitudinal astronaut study^66^ (*n* = 17, mean age = 46·8) compared *n* = 10 flying long‐duration versus *n* = 7 short‐duration. Pre‐flight, no group differences were found in deep or periventricular WMH volumes. In unadjusted pre‐ versus post‐flight comparisons, no deep WMH changes were found but periventricular WMH increased (0·21 mL [SD = 0·19 mL]; 39% increase) in the long‐duration versus 0·02 mL (SD = 0·05 mL) in the short‐duration group, *p* = 0·01. At 1‐month follow‐up, these WMH increases had partly reversed (65% reduction).

Two further longitudinal astronaut studies assessed pre‐ and post‐spaceflight PVS differences. MRI after 6‐month spaceflight (*n* = 37 cases, *n* = 11 controls, mean age = 48) detected increased post‐flight PVS volumes (pre‐flight PVS mean in space‐station group = 1152 mm3 [SD = 559] versus post‐flight = 1435 mm3 [SD = 698], *p* < 0·001; no change in controls).^69^ The second pre‐ and 6‐month post‐spaceflight study (15 cases, 11 controls) detected increasing PVS volumes in novices (estimate for total PVS change (mm^3^/cm^3^) = 0·16; [SE = 0·06]; *p* = 0·02) with no change in PVS number. Experienced astronauts’ PVS volumes decreased post‐flight.^70^ All high‐altitude exposures were occupational.

### 3.4 Narrative synthesis

We narratively reviewed contact sports, lead, military, and pesticide/fertilizer exposures as estimates were too varied or few to meta‐analyze.

#### 3.4.1 Contact sports

We identified three case‐control studies assessing relationships between professional contact sports and SVD, *n* = 629.^71^, ^72^, ^73^ There were no WMH differences in fighters (boxers, mixed martial artists, martial artists): 118/499 (23·6%) versus controls 12/62 (19·4%), *p* = 0.52, mean age = 30.^71^ Similarly, in retired contact sports athletes and age‐matched non‐contact controls, there were no differences in WMH prevalence 12/21 (57·1%) versus 11/21 (52·4%).^73^ Neither study detected differences in microbleed prevalence/number.^71^, ^73^ In a selected population of former American football players with cognitive deficits versus healthy controls (*n* = 26),^72^ unadjusted but no differences in vascular risk factors, total and deep WMH volumes were higher in the player group (mean = 8·13 mL vs. 2·38 mL) but periventricular WMH volumes did not differ, mean age = 61·8. All sport exposures were occupational.

#### 3.4.2 Lead

We identified two studies assessing SVD associations with lead,^74^, ^75^ *n* = 423. In 61 current lead workers, 14/61 (23%) had WMH, mean age = 40.^74^ A longitudinal study comparing former lead manufacturers with controls (mean age = 60·8; *n* = 309 one MRI; 362 two MRI; mean exposure‐imaging interval = 14 years) found that cumulative lead dose was not associated with Cardiovascular Health Study WMH score change.^75^ All lead exposures were occupational.

#### 3.4.3 Military

We identified 10 studies (*n* = 18,893) assessing SVD in military occupations (Supplementary Table 1H).^76^, ^77^, ^78^, ^79^, ^80^, ^81^, ^82^, ^83^, ^84^, ^85^ These studies were not meta‐analyzable due to heterogeneously reported estimates. In two studies, mostly unadjusted, VaD prevalence was not high considering the populations’ ages: 3,931/168,111 (2·3%) of former army veterans >65 years had VaD;^76^ 7/60 (11·6%; mean age = 64) had vascular MRI or VaD diagnosis.^77^ There were conflicting microbleed findings in three studies of military members with traumatic brain injury (TBI) in their 30s.^78^, ^79^, ^81^ Two studies detected microbleeds in 43/603 (7·6%) active duty members (mean age = 33·8) with chronic TBI^78^ and in 60/834 (7·2%) TBI participants versus 0/42 controls (OR = 6·64, 95% CI = 0·4‐198·2),^81^ whereas one study reported no microbleeds in veterans with chronic blast‐related TBI.^79^ WMH and TBI/blast exposure were not associated in one study^82^ but were associated in three.^80^, ^81^, ^84^ Two studies showed possible TBI‐PVS associations.^81^, ^83^ In 107 veterans, half with versus half without TBI, mean age = 68·5, pooled mean WMH volume = 5·3 mL (SD = 6·0) and the TBI group had more CSO‐PVS (20·3 [SD = 9·7]) vs 14·1 [SD = 8·4]; *p* < 0·001).^85^

#### 3.4.4 Pesticides/fertilizers

A single population‐based cohort study (*n* = 8,623, mean age = 69·4)^86^ found that incident VaD risk was higher in individuals occupationally exposed to pesticides/fertilizers (OR = 2·05; 95% CI = 1·03‐3·85) but not plastic/rubbers (OR = 1·75, 95% CI = 0·57‐4·45).

#### 3.4.5 Miscellaneous solvents

Four studies assessed miscellaneous solvent exposure associations with SVD,^87^, ^88^, ^89^, ^90^ *n* = 251, mean age 38 years. Populations had been exposed to toluene, xylene, white spirit/thinner, trichloroethylene, and industrial alcohols. The SVD prevalence was 25% in populations exposed to domestic solvent inhalation,^88^ 28%‐100% in populations exposed to common industrial solvents,^87^, ^89^ and 35% in chronic solvent abusers of laquer thinner containing toluene.^90^ Two studies involved deliberate abuse;^88^, ^90^ two involved occupational exposures.^87^, ^89^

#### 3.4.6 Broad occupational categories

We identified nine studies^91^, ^92^, ^93^, ^94^, ^95^, ^96^, ^97^, ^98^, ^99^ (*n* = 15,118) reporting VaD incidence/prevalence across broad‐ranging occupations, mostly retired (Supplementary Table 1L). Four studies showed possible higher VaD prevalence among retired farmers.^93^, ^95^, ^97^, ^99^ However, this diminished after adjusting for vascular risk factors in three studies.^93^, ^95^, ^99^ No farming associations with dementia were identified in three further studies.^91^, ^94^, ^98^

For blue‐collar workers, four studies reported no VaD associations ^91^, ^93^, ^98^, ^99^ and one study reported an unadjusted association (OR = 5·22 [95% CI = 2·36‐11·54]).^97^ For white‐collar workers, two studies found no VaD associations, ^91^, ^99^ while another reported an unadjusted association [OR = 2·99 (95% CI = 1·50‐5·95)].^97^ One study found VaD associations in retired transportation/logistics workers, but this diminished after adjusting for vascular risk.^94^ One study found no VaD association with housework.^97^ One small retrospective study reported unadjusted associations between possible VaD in retired electronic/electrical workers (OR = 3.0 [95% CI = 1·2‐7·6]), laborers (OR = 6·3 [95% CI = 1.0‐39·2]), and food/beverage processors (OR = 7·3 [95% CI = 2·0‐27·3]); *n* = 195 with dementia, of which 59/195 had VaD; 229 controls.^96^

### 3.5 Case‐control studies

See Supplementary Table 5.

### 3.6 Longitudinal studies

We describe nine longitudinal imaging studies.^16^, ^18^, ^25^, ^46^, ^62^, ^69^, ^70^, ^75^, ^84^ in Supplementary Table 6.

### 3.7 Meta‐regression

We were only able to perform meta‐regression for age for carbon monoxide studies since this was the only exposure containing >10 studies. Age did not explain SVD variance (slope = 0·004 [SE = 0·04]; *p* = 0·93; Supplementary Figure 1). Meta‐regression for exposure duration or substance concentration was not possible as few studies reported this per category, for example, 23/29 carbon monoxide studies reported ongoing exposure; three were unreported.

### 3.8 Risk of bias

Most studies (65/85) reported unadjusted frequencies with high risk of bias. Where studies adjusted for confounders, this was mainly done for age but rarely for educational attainment, socioeconomics, or vascular diagnoses. The main biases were non‐adjustment for confounders and blinding. See Supplmenetary Table 3. Heterogeneity varied 0%‐79%.

## 4 DISCUSSION

This is the first systematic review and meta‐analysis to examine occupational and domestic exposures in relation to SVD and VaD. We found a high prevalence of radiological SVD features in populations exposed to carbon monoxide and carbon disulfide, but not in populations exposed to lead, contact sports, or diving. We found a moderate SVD prevalence in populations exposed to high‐altitude work, with possible associations between longitudinal WMH and PVS enlargement in astronauts in small unadjusted studies. There was insufficient available evidence to confirm or refute associations between radiological SVD features and exposure to 1,2‐dichloroethane, toluene, or other solvents. Pesticide/fertilizer exposure carried a two‐fold VaD risk. Associations between military work and VaD were inconclusive. Possible farming and transport associations with VaD were reported in several studies but these disappeared adjusting for vascular risk factors. It is unclear whether vascular risk factors are mediators in SVD development in these occupations. We were unable to determine whether higher SVD risk or greater SVD severity exists in exposed versus unexposed populations due to insufficient evidence. We did not find studies assessing lacunar stroke associations. Other SVD features apart from WMH were understudied.

The included studies had limitations. Most were observational. There were insufficient case‐control studies per category for pooled meta‐analysis. Therefore, our meta‐analyses were mainly restricted to pooling SVD prevalence in observational cohorts. Meta‐analyzed sample sizes were small. Most studies reported unadjusted findings and did not adjust for age, vascular risk factors, alcohol intake, or socioeconomic status. Consequently, we were unable to perform meta‐regression to assess their influence. We detected high risk of bias. Findings should be interpreted with caution, considering most results were unadjusted and blinding was unreported. Many studies were older and used CT. Exposure concentrations were poorly reported overall (Supplementary Table 1), likely due to the methodological issues involved in quantifying exposures. As a result, it was not possible to quantify the contribution of recency, quantity, or duration of exposure to our findings, for example, chronic low‐level versus, acute exposures. Finally, we did not identify any studies examining the effects of exposure on novel SVD markers such as microinfarcts so the prevalence of these and other SVD features should be a target for future research.

Our review had limitations. Our research question is underexplored in both the SVD and occupational literature, so we may have omitted some hazardous substances or workplaces. However, our search was based on occupational hazards identified from UK/EU regulators. Due to few case‐control studies per category, we included exposed cases only in our meta‐analyses to enable pooling of prevalence data with observational studies of entire exposed cohorts. However, we also narratively synthesized case‐control studies separately. Suggested reasons for moderate‐high heterogeneity include the spectrum of exposure concentrations, durations, and exposure‐imaging intervals. Most studies reported SVD prevalence only. Comparing prevalence rates with the general population is challenging because few published studies report SVD prevalence in adults in their 40s.

Our systematic review and meta‐analysis build on previous work establishing links between early life factors and SVD.^3^, ^5^ It is unclear whether occupation may have interactive, versus, independent additive effects with socioeconomics and education on later‐life risk of brain vascular disease.

See Supplementary Table 7 for typical exposure settings for included substances. Carbon monoxide exists in domestic, occupational, and environmental settings. There is growing evidence of an air pollution‐dementia link.^100^, ^101^ We are aware of few studies specifically assessing links between air pollution and SVD ^102^, ^103^ or VaD.^104^, ^105^, ^106^ A recent study ^107^ found that hypertension increased susceptibility but did not modify all‐cause incident dementia associations with air pollution, suggesting alternative risk mediators. Our findings propose SVD as a potential mediator of this risk, since part of our analysis reports on inhaled environmental exposures. However, we did not assess air pollution per se and almost no studies reported vascular risk factors, so further work is needed to determine air pollution associations with SVD and VaD. Carbon monoxide has > 200 times affinity for hemoglobin versus oxygen,^108^ deoxygenating brain tissue, providing biological plausibility for an association between air pollution and SVD. A community‐based study found higher exhaled carbon monoxide associated with higher WMH burden, silent infarcts, and future stroke.^109^ Carbon disulfide is used during rubber and rayon processing. It has been understudied but may be associated with systemic vascular dysfunctions including impaired lipid clearance, fibrinolysis, and myocardial toxicity.^110^ Astronaut studies have proposed that anti‐gravity brain shifts may obstruct CSF‐interstitial fluid channels, promoting PVS dilatation.^69^ Clinical and imaging parallels between toxic leukoencephalopathy, well‐described in occupational literature,^111^ and SVD, require further exploration.

Diffuse toxic effects of exposures to cerebral white matter may be even more prevalent when normal‐appearing white matter (NAWM) is assessed for subvisible features of damage to white matter microstructure,^112^, for example, using diffusion tensor imaging (DTI). A greater focus on NAWM using DTI in epidemiological studies could offer early and mid‐life clues to dementia pathogenesis that are currently being overlooked.

## 5 CONCLUSIONS

Occupational exposure to hazardous substances and settings may be associated with SVD and VaD. Our findings emphasize the clinical importance of occupational history. There is insufficient evidence in this analysis to support changes in clinical approaches to occupational or vascular health. However, our findings have implications for research in SVD, dementia, stroke, and occupational health. Much more work is needed to determine safe thresholds of exposure and the spectrum of clinical implications. The etiology of both SVD and VaD is poorly understood and there are gaps in our understanding of how early and midlife exposures may influence risk. Since the present analysis demonstrates a high prevalence of SVD features and VaD in individuals with specific exposures, the concentration thresholds and durations of environmental exposures should now be explored to address these gaps, and should extend beyond the exposures reported in the current literature. Once implications become clearer, regulators may need to revisit workplace health and safety guidelines.

It is possible that vascular risk factors have a mediating effect on SVD‐occupational relationships. Early exposures may act as a precursor, creating an early to midlife vulnerability to the development of SVD and increased susceptibility to the effects of vascular risk factors in later life. Moreover, many hazardous substances carry increased risk of systemic vascular disease^111^ **;** therefore, it is possible that certain substances are common denominators in the etiology of both SVD and vascular risk factors. Occupational exposures in younger versus older brains need further exploration. To address these gaps, we need large epidemiological studies assessing dose‐response relationships across the life‐course.

## CONFLICTS OF INTERESTS STATEMENT

U.C., Y.C., F.N.D., and J.M.W. hold academic grants from government and charitable funding agencies. A.B. has nothing to declare. Author disclosures are available in the supporting information.

## Supporting information

Supporting Information

Supporting Information
