# Supplementary material for: Occupational and domestic exposure associations with cerebral small vessel disease and vascular dementia: A systematic review and meta‐analysis
Source: Alzheimers Dement. 2024 Jan 25;20(4):3021–33. doi: 10.1002/alz.13647 (PMC11032565; doi:10.1002/alz.13647)
Supplement: Supplementary file 1 — Supporting Information [file ALZ-20-3021-s001.docx]

# Occupational and domestic exposure associations with cerebral small vessel disease and vascular dementia: a systematic review and meta-analysis

Corresponding author: Joanna Wardlaw

UK Dementia Research Insittute and Centre for Clinical Brain Sciences, University of Edinburgh, Chancellor’s Building, 49 Little France Crescent, Edinburgh, UK, EH16 4SB

Email: joanna.wardlaw@ed.ac.uk

Tel: +44 131 465 9599

**Supplementary text, tables, and figure**

Supplementary Text 1: Search Strategy (pg.2)

Supplementary Table 1: Summary of included studies (pg. 5)

Supplementary Table 2: Terms used to describe radiological features of SVD (pg. 25)

Supplementary Table 3: Risk of bias assessment (pg. 26)

Supplementary Table 4: Summary of studies and sample sizes included in meta-analyses vs narrative synthesis according to substance/occupational exposure (pg. 28)

Supplementary Table 5: Summary of case control studies (pg.29)

Supplementary Table 6: Summary of longitudinal study findings (pg. 31)

Supplementary Figure 1 Meta-regression of SVD prevalence in carbon monoxide studies adjusting for age (pg. 34)

Supplementary Table 7: Typical exposure settings for each of the substances studied (pg. 35)

## Supplementary Text 1

Database: Embase Search Strategy: ---------------------<1974 to 2023 Week 8> ---------------

1 exp Cerebrovascular disease/ and (White matter lesion/ or Vascular lesion/)

2 exp Lacunar stroke/ or exp Brain Stem Infarction/ or exp Perivascular space/ or exp White matter lesion/ or exp Leukoaraiosis/

3 (small vessel disease* or small vessel cerebrovascular disease* or SVD or small vessel cerebrovascular disorder).tw.

4 (subcortical infarct* or subcortical cystic infarct* or subcortical stroke or deep infarct* or silent stroke or silent infarct* or silent cerebral infarct* or silent brain infarct* or (perforat* adj3 infarct*)).tw.

5 (lacun* or small deep brain infarct or small deep brain h?emorrhage).tw.

6 (leu?oaraiosis or leu?oencephalopathy).mp.

7 (perivascular space* or peri-vascular space* or Virchow Robin space* or etat crible).tw.

8 ((Microinfarct* or microscopic infarct* or microangiopath*) and (brain or cerebr*)).tw.

9 ((small vessel* or small) adj3 (infarct* or stroke*)).tw.

10 ((Micro-bleed* or microbleed* or micro h?emorrhage* or microh?emorrhage* or (dotlike and h?emosid*)) and (brain or cerebr* or cerebral small vessel disease*)).tw.

11 exp Multiinfarct Dementia/

12 ((subcortical adj3 dementia) or vascular cognitive impairment or vascular dementia or small vessel dementia).tw.

13 (Binswanger* or (subcortical adj3 encephalopathy) or (subcortical adj2 leu?oencephalopathy)).tw.

14 exp Occupational Disease/ or exp Occupational Exposure/ or exp Occupational Hazard/ or exp Occupation/ or exp Workplace/ or exp Nonmedical Occupations/ or exp Organic Solvent/ or exp Brain Toxicity/

15 exp arsenic poisoning/ or exp cadmium poisoning/ or exp organophosphate poisoning/ or exp gas poisoning/ or exp Welding/ or exp Coal Mining/

16 (occupation* or workplace* or worker* or industrial).tw.

17 (irritant or corrosive or poison* or hazardous or noxious or caustic).tw.

18 (engineer or electrician or motor vehicle repair* or mechanic or truck or lorry or train or automobile or airplane or aeroplane or airport or fuel or oil or refinery or petrol* or petrochemical or diesel or kerosene or gasoline or exhaust or lubrica* or fumigant or fumigat* or offshore or oil rig).tw.

19 (agricultur* or farm* or pesticide or feed additive or fertiliser or soil improver or insecticide or fungicide or organophosphate).tw.

20 (print* or paint* or lacquer or adhesive or sealant or glue or spray or isocyan* or ink or primer or dye).tw.

21 (woodwork* or carpent* or joiner or glazier or quarry or quarries or quartz or varnish or dust* or resin or binder or formaldehyde or silic* or putty or sand or foundry or stoneworker or pickling paste or metalwork* or coolant or glycol or miner or mining or coal or solder* or weld* or fume* or steelworker or electronic or microelectronic or plumb* or roof* or fitter or flooring or tile or tiling or vinyl or toxic metal or heavy metal or cyanide or arsenic or cadmium or manganese or "lead exposure" or mercury or chromium or chromate or beryllium or aluminium or copper or zinc or organic tin).tw.

22 (cater* or beaut* or hairdress* or print* or dry?clean* or textile or rayon or viscose or acrylic or furniture or upholst* or shoe or leather or food industry or degreas* or ammoni* or detergent* or baking or baker or cleaning product or cleaner or cleanser or disinfect* or fluorin* or chlorin* or polychlorinated or dichloromethane* or sodium hypochlorite or lye* or sodium hydroxide).tw.

23 (gas or vapo?r or volatile or mist or fire* or flame or flamm* or combust* or explosive* or inhal* or tar or carbon monoxide or carbon disulphide or carbon disulfide or hydrogen sulphide or hydrogen sulfide or hydrogen sulphide or solvent* or hydrocarbon or toluene or benzene or turpentine or white spirit* or paint?thinner or ethylbenzene or styrene or xylene or methanol or carbon tetracholoride or polyvinyl or ethylenediaminetetraace* or polymer or perchloroethylene or trichloroethylene or bromide or bromin* or methylene or ethylene glycol or isopropranol or acetone or polyurethane or phthalate or paraffin).tw.

24 (insulation or asbestos* or fibreglass or shipyard or shipbuild* or dockyard or marine or builder or construction or "manual labo?r" or cement or rubber or brick* or ceramic).tw.

25 1 or 2 or 3 or 4 or 5 or 6 or 7 or 8 or 9 or 10 or 11 or 12 or 13

26 14 or 15 or 16 or 17 or 18 or 19 or 20 or 21 or 22 or 23 or 24

27 25 and 26

28 animal/ not human/

29 27 not 28

30 letter/ or case study/ or editorial/ or conference abstract/ or "electronic health".mp. or "electronic database*".mp. or "occupational therapy".mp.

31 29 not 30

Database: Ovid MEDLINE(R) Search Strategy: -------------<1946 to March Week 1 2023> -----------

1 exp Cerebral Small Vessel Disease/ or exp Stroke, Lacunar/ or exp Leukoaraiosis/ or exp Brain Stem Infarctions/

2 (small vessel disease* or small vessel cerebrovascular disease* or SVD or small vessel cerebrovascular disorder).tw.

3 (subcortical infarct* or subcortical cystic infarct* or subcortical stroke or silent stroke or silent infarct* or silent cerebral infarct* or silent brain infarct* or (perforat* adj3 infarct*)).tw.

4 *lacun*/

5 (white matter hyperintens* or white matter disease* or white matter lesion* or white matter change* or white matter damage or WML or WMH or leu?oaraiosis or leu?oencephalopathy).tw.

6 (perivascular space* or peri-vascular space* or Virchow Robin space* or etat crible).tw.

7 "white matter".mp. or "small subcortical infarct".tw.

8 ((Microinfarct* or microscopic infarct* or microangiopath*) and (brain or cerebr*)).tw.

9 ((small vessel* or small) adj3 (infarct* or stroke*)).tw.

10 ((Micro-bleed* or microbleed* or micro h?emorrhage* or microh?emorrhage* or (dotlike and h?emosid*)) and (brain or cerebr* or cerebral small vessel disease*)).tw.

11 exp Dementia, Vascular/

12 ((subcortical adj3 dementia) or vascular cognitive impairment or vascular dementia or small vessel dementia).tw.

13 (Binswanger* or (subcortical adj3 encephalopathy) or (subcortical adj2 leu?oencephalopathy)).tw.

14 exp Occupational Exposure/ or exp Occupations/ or exp Workplace/ or exp Occupational Diseases/ or exp Occupational Groups/ or exp Solvents/ or exp Neurotoxicity Syndromes/

15 exp Gas Poisoning/ or exp Heavy Metal Poisoning/ or exp Heavy Metal Poisoning, Nervous System/ or exp Organophosphate Poisoning/ or exp Welding/ or exp Coal Mining/

16 (occupation* or workplace* or worker* or industrial).tw.

17 (irritant or corrosive or poison* or hazardous or noxious or caustic).tw.

18 (engineer or electrician or motor vehicle repair* or mechanic or truck or lorry or train or automobile or airplane or aeroplane or airport or fuel or oil or refinery or petrol* or petrochemical or diesel or kerosene or gasoline or exhaust or lubrica* or fumigant or fumigat* or offshore or oil rig).tw.

19 (agricultur* or farm* or pesticide or feed additive or fertiliser or soil improver or insecticide or fungicide or organophosphate).tw.

20 (print* or paint* or lacquer or adhesive or sealant or glue or spray or isocyan* or ink or primer or dye).tw.

21 (woodwork* or carpent* or joiner or glazier or quarry or quarries or quartz or varnish or dust* or resin or binder or formaldehyde or silic* or putty or sand or foundry or stoneworker or pickling paste or metalwork* or coolant or glycol or miner or mining or coal or solder* or weld* or fume* or steelworker or electronic or microelectronic or plumb* or roof* or fitter or flooring or tile or tiling or vinyl or toxic metal or heavy metal or cyanide or arsenic or cadmium or manganese or "lead exposure" or mercury or chromium or chromate or beryllium or aluminium or copper or zinc or organic tin).tw.

22 (cater* or beaut* or hairdress* or print* or dry?clean* or textile or rayon or viscose or acrylic or furniture or upholst* or shoe or leather or food industry or degreas* or ammoni* or detergent* or baking or baker or cleaning product or cleaner or cleanser or disinfect* or fluorin* or chlorin* or polychlorinated or dichloromethane* or sodium hypochlorite or lye* or sodium hydroxide).tw.

23 (gas or vapo?r or volatile or mist or fire* or flame or flamm* or combust* or explosive* or inhal* or tar or carbon monoxide or carbon disulphide or carbon disulfide or hydrogen sulphide or hydrogen sulfide or hydrogen sulphide or solvent* or hydrocarbon or toluene or benzene or turpentine or white spirit* or paint?thinner or ethylbenzene or styrene or xylene or methanol or carbon tetracholoride or polyvinyl or ethylenediaminetetraace* or polymer or perchloroethylene or trichloroethylene or bromide or bromin* or methylene or ethylene glycol or isopropranol or acetone or polyurethane or phthalate or paraffin).tw.

24 (insulation or asbestos* or fibreglass or shipyard or shipbuild* or dock or dockyard or marine or builder or construction or "manual labo?r" or cement or rubber or brick* or ceramic).tw.

25 1 or 2 or 3 or 4 or 5 or 6 or 7 or 8 or 9 or 10 or 11 or 12 or 13

26 14 or 15 or 16 or 17 or 18 or 19 or 20 or 21 or 22 or 23 or 24

27 (25 and 26) or "chronic toxic encephalopathy".mp.

28 animal/ not human/

29 27 not 28

30 letter/ or case study/ or editorial/ or conference abstract/ or "electronic health".mp. or "electronic medical".mp. or "electronic database*".mp. or "occupational therapy".mp.

31 29 not 30

## Supplementary Table 1. Summary of included studies

## 1A: Carbon monoxide

| **Study**  **(Year)** | **Population** | **Study design** | ***n*** | **Mean age** | **MRI vs CT** | **Imaging abnormalities and location** | **Duration/concentration of exposure** | **Exposure-imaging interval** | **SVD prevalence** |
| --- | --- | --- | --- | --- | --- | --- | --- | --- | --- |
| Chang 1992 | Patients who had relapse of neuropsychiatric symptoms 4-9 weeks after initial recovery from acute CO intoxication/unconsciousness due to leaks in underfloor heating systems | Mostly cross-sectional: 4 had longitudinal imaging | 15 | 50 | MRI | Bilateral symmetric confluent high signal intensity in the periventricular white matter and centrum semiovale in all (n = 15). In 9/15, there was diffuse uniform distribution of the lesions throughout the deep white matter, with no predilection sites. In 6/15, the lesions were more extensive and prominent in the frontal lobes. Associated focal hemorrhage in the right globus pallidus and left putamen in n=1. WMH decrease seen in 3/4 patients with follow-up MRI, increase in n=1. In n=1, WML markedly reduced and the lesions of the globus pallidus disappeared on the 1-month follow-up study, but WML remained as small foci in the centrum semiovale on the 9-month follow-up study | Not clear but all acute exposure | 4-9 weeks after exposure. In 4 patients with longitudinal imaging, follow-up MRI occurred 1-9 months after initial MRI | 100% (15/15) |
| Chang 2010 | Patients admitted to hospital with delayed neuropsychiatric syndrome after acute CO poisoning according to family, all patients received piracetam or bromocriptine or both plus hyperbaric oxygen therapy | Cross-sectional observational study | 9 | 34.3 | MRI | White matter changes remained evident on MRI after 8-40 sessions of hyperbaric oxygen therapy - brain MRI scans revealed generalized diffuse white matter demyelination in 9/9 - T2-weighted and FLAIR images showed bilateral areas of confluent high signal intensity in the periventricular white matter and centrum semiovale in 9/9. 6/9 had necrosis of the bilateral globus pallidi. MRI repeated as the symptoms improved after serial sessions of hyperbaric O2: no interval improvement seen. Previously necrotic globus pallidi became partially collapsed. | Not clear | At least 30-60 days (time from suffering acute CO poisoning to presenting with delayed neurospychiatric symptoms requiring hospital admission) | 100% (9/9) |
| Chen 2005 | Patients with delayed encephalopathy after acute CO poisoning | Cross-sectional observational study | 46 | 55.8 | both | 38/46 had CT scan diffuse low density abnormalities in bilateral cerebral white matter, bilateral or unilateral globus pallidus or basal ganglia areas. MRI scans showed cerebral white matter demyelination mainly around the ventricles, with high signal intensity in T(2)-weighted and equal or low signal intensity in T(1)-weighted as well as lesions in hippocampus and brain stem | Ongoing | Not clear | 83% (38/46) |
| Choi 1993 | Patients admitted to hospital with CO poisoning, majority of whom had been unconscious | Mostly cross-sectional mostly but longitudinal imaging in 36 patients with delayed or prolonged neurologic sequelae | 129 | 48.2 | CT | Most common finding, in 42/129, was low-density in the cerebral white matter particularly in frontal areas. Second characteristic feature, in 33/129 patients, was low-density in both globus pallidi. Abnormal CT findings tended to increase in accordance with the duration of unconsciousness during acute CO poisoning. Follow-up CT showed no interval change in 14/25 and 8 revealed an 'aggravating pattern' with cortical atrophy | Not clear | Within 3 days (n=123);within 14 days (n=6). Follow-up CT for delayed neurological sequelae subset, 3-8 months (n=23); 18-26 months (n=2) | 33% (42/129) |
| Durak 2005 | Chronic stage of accidental CO poisoning, all comatose initially and all awoke within 1-7 days on normobaric oxygen. Exposed to indoor burning of wood/coal in a brazier or stove within past 10 years. Eligible participants identified from medical records. | Cross-sectional observational study | 16 | 32.2 | MRI | In all patients there were varying degrees of bilateral symmetric WMH that were significant in the centrum semiovale, with relative sparing of the temporal lobes and anterior parts of the frontal lobes. Bilateral globus pallidus lesions were seen in n=3. | Not clear | Routine MRI 1-10 years (mean 3.4 years after exposure) | 100% (16/16) |
| Fukuhara 1996 | 15 acute CO poisoning cases vs 16 age- and sex-matched controls | Case-control study | 31 | 59.1 | Both | 7/15 exposed cases had bilateral lesions in the globus pallidus, 4/15 had lesions in the parietotemporooccipital lobe bilaterally, and n=5 had multiple deep WML. Control group brain scan findings not reported | Not clear | Not clear | 67% (10/15) |
| Hao 2017 | Moderate and severe acute CO poisoning | Cross-sectional observational study | 95 | 40.2 | both | All had lesions in unilateral or bilateral cerebral cortex, bilateral basal ganglia, cerebral white matter around bilateral ventricles or bilateral centrum semiovale, cerebral cortex and subcortical involvement. CT showed normal or low density shadow. MRI showed that the lesion T(1)WI presented slightly low or equal signal, T(2)WI and FLAIR sequences showed equal, a slightly higher or high signal; DWI sequence showed slightly higher or high signal | Unclear | Unclear | 100% (95/95) |
| Hsiao 2004 | Delayed encephalopathy after acute CO poisoning - all had altered consciousness and had received high flow oxygen or hyperbaric oxygen therapy at acute presentation | longitudinal observational study | 12 | 54.5 | MRI | Initial MRI multiple hyperintensities on T2 in the subcortical white matter and basal ganglia, mostly in the globus pallidus, and to a lesser degree in the putamen, and caudate, and midbrain. Thalamus was spared in all. Subcortical WM lesions symmetric in n=8 and asymmetric in n=1. Follow-up brain MRI showed a steady improvement (n=6), revealing prominent improvement in subcortical WM and GP lesions in all 6 patients. Improvememt in putamen and caudate lesions in n=2, globus pallidus lesion improved in another patient | All had a clear history of exposure and/or high COHb level | First MRI within 1 week, second MRI not clear | 100% (12/12) |
| Kim 1980 | Patients with acute carbon monoxide poisoning | Cross-sectional observational study | 9 | 38.3 | CT | The most common CT feature was symmetrical, bilateral, low density basal ganglia abnormalities, more dense than CSF in the early stage and as well-defined and more lucent lacunae in the late stage. A second feature was diffuse symmetrical white matter low density areas in 3/9 cases, poorly-defined and faintly lucent areas in the periventricular white matter in the early phase, then better-defined and more lucent later. All five patients who had scans ≥6 weeks post-exposure showed bilateral symmetrical lacunae in the basal ganglia. The lesions were well defined and more lucent with density similar to CSF. | Not clear | same day to 3 months | 100% (9/9) |
| Kim 2003 | Consecutive patients with delayed encephalopathy of CO intoxication with relapse of neuropsychiatric symptoms after initial recovery, majority exposed to gas leakage from under-floor heating system | Cross-sectional observational study | 5 | 63.2 | MRI 1.5T | In 5/5, both T2-weighted images and DWIs showed the white matter lesions as bilateral, diffuse, confluent areas of hyperintensity in the periventricular white matter and centrum semiovale. On ADC maps, these lesions were isointense, with focal areas of hypointensity (n = 4) or diffuse hypointensity (n = 1) | Unclear | 25-95 days | 100% (5/5) |
| Kim 2017 | Acute CO poisoned patients, all with mental status change, all of whom underwent hyperbaric oxygen therapy or oxygen therapy | Cross-sectional observational study | 7 | 43 | MRI | All seven patients (7/7, 100%) showed restricted diffusion of the lesions on ADC maps and bilateral involvement of globus pallidus. Cerebral cortex, cerebral white matter, cerebellum, hippocampus, amygdala, splenium of corpus callosum, midbrain and insula were also involved. Especially, periventricular white matter was predominantly involved in the supratentorial space. n=4 had small foci of dark signal intensity of petechial haemorrhage in the bilateral globi pallidi. | Mean COHb= 22.3%, range 8.3 to 34.8% | <7 days | 100% (7/7) |
| Lee 1994 | Accidental CO poisoning: exposure to leaked underfloor heating coal stoves during sleep, with subsequent development of neurological sequelae.  group 1 (progressive type)  group 2 (delayed relapsing type) | Longitudinal in 8/31, cross-sectional in remainder | 31 | 50.6 | CT | 3/8 patients in group 1 had a normal CT scan, n=1 had white matter low-density (WMLD) lesions, one had bilateral globus pallidus low-density (GPLD) lesions, and three had both WMLD and GPLD lesions. 7/23 in group 2 had normal CT at the onset of the sequelae (n=6 improved and n=1 no change), n=12 had WMLD lesions (n=7 improved, n=1 no change), n=1 had a unilateral GPLD lesion and n=2 had bilateral GPLD lesions, and n=1 had both WMLD and bilateral GPLD lesions (showed no change). Follow-Up Brain CT Scans in n=8; all revealed progression of the changes and/or new lesions. N=2 had abnormal findings only on follow-up CT scans. 5/8 of group 1 and 16/23 of group 2 had white matter abnormalities = 21/31 | The mean duration (+SD) of initial coma of group I  (9.8days) was significantly longer than that of group 2 (2 days).The mean (kSD) of CO hemoglobin level was not significantly different between the groups (group I: 28.1 +- 7.0%; group 11: 29.1 +- 5.6%). | 1 week | 26% (8/31) |
| Liu 2001 | Patients with delayed encephalopathy after acute CO poisoning | Cross-sectional observational study | 20 | 41.5 | Both | Diffuse low density was observed within bilateral cerebral white matter, prominently within bilateral or unilateral globus pallidus or basal ganglia areas. The MRI showed that brain lesions were bilateral symmetric punctate, spotty or confluent foci within periventricular white matter and centrum semiovale, which showed high signal intensity in T2-weighted and low signal intensity in T1-weighted. The chronic ischemia changes were found in the globus pallidus or basal ganglia areas. Cerebral cortex lesions were observed occasionally. | Unclear | Unclear | Not clear |
| Liu 2020 | Delayed encephalopathy after CO poisoning in patients who received hyperbaric oxygen treatment. (1) A clear history of CO poisoning resulting in coma in the previous 1-2 months; (2) normal or nearly normal performance in the interim; (3) clinical features of acute dementia or full-brain damage, including advanced neurological deficits, psychiatric symptoms, pyramidal system disorders, and extrapyramidal manifestations, coupled with peripheral and cranial nerve damage. Only patients with prominent leukoencephalopathy but no subclinical leukoencephalopathy were recruited. | Retrospective medical record review | 20 | 61 | MRI | 7/20 (35%) with high signal intensity in the bilateral white matter, n=2 (10%) with basal ganglia lesions, and n=11 (55%) with basal ganglia lesions accompanied by the periventricular white matter and semielliptical central lesions. | Clear history of CO poisoning at presentation but not clear | MRI performed on first day of hospitalisation | 55% (11/20) |
| Matsushita 1996 | Patients with CO poisoning and acute disturbance of consciousness | Retrospective mostly cross-sectional study, n=9 had longitudinal imaging | 13 | 38 | MRI | White matter lesions seen on T2W in n=8 patients, sequential studies showed a tendency of regression of hyperintense lesions. N=5 had lesions in bilateral globus pallidi on T2-w images and hypointensity on T1-w imaging. | Not clear but conscious disturbance lasted from a few hours to 10 days | Not clear | 62% (8/13) |
| Mimura 1999 | Carbon monoxide poisoned patients | Cross-sectional observational cohort study | 156 | 69.2 | MRI (129) | Lacunar infarction in 52.7% (68 cases), globus pallidus lesion in 37.9% (40 cases), | Not clear | 33 years | 44% (68/156) |
| Miura 1985 | Hospitalised patients exposed to CO with coma following accidental or intentional inhalation of natural gas, smoke, or automobile exhaust | Longitudinal observational study (part cross sectional) | 60 | 32.5 | CT | The most common finding, seen in 21/60, was symmetric and diffuse low density in the cerebral white matter, which was more advanced in the centrum semiovale and varied in degree from slight to severe; severe in 10/21. In 18 patients there was a symmetric, bilateral, round low-density lesion in the globus pallidus. Follow-up CT scans were obtained in n=15 1-10 days after the first CT. Resolution of white-matter changes occurred in n=3, no change in remainder. The low densities of the globus pallidus decreased in n=1, increased in n=5, and decreased but later improved in n=1. | Not clear but all patients comatose when found | Of pts with abnormal scans, first was CT within 24hrs after patient found in n=12; within 48hrs in n=6; from 4-15 days in n=5 | 35% (21/60) |
| Nah 2020 | CO poisoned patients attending the emergency department who had undergone HBO therapy | Cross-sectional observational study | 154 | 40.7 | MRI | Acute brain lesions observed on DWI and ADC MRI were observed in 49/154 patients (31.8%), mainly deep nucleus, cerebellum, white matter, cortex. | COHb was 6.0 (2.8–11.8) in Non-delayed neurological sequelae group and 9.3 (4.2–16.7) in Delayed Neurological Sequelae group | 2 days | 32% (49/154) |
| O'Donnell 2000 | Consecutive acute CO-poisoned patients, all of whom were unconscious at presentation and all received hyperbaric oxygen | Longitudinal in 5/19; cross sectional in remainder | 19 | 38.7 | MRI 0.5T | 13/19 had any hyperintensity involving subcortical regions. Abnormalities were found in the following areas: globus pallidus (n=12); other basal ganglia [n=5: entire lentiform (globus pallidus and putamen), putamen alone, caudate nucleus, thalamus]; white matter (n=6: periventricular, subcortical, other); cerebral cortex (n=5), either localized or general; medial temporal lobe in the region of the hippocampus (n = 4). Normal appearances were seen on the initial MR in 7/19, one of these became abnormal on subsequent imaging. Progression of abnormalities occurred in 4/5 who had more than one MR. | Mean COHb 34.4 (13.1) | 35.6 hours (range 6-126 hours) following acute presentation at referring centre | 68% (13/19) |
| Otubo 2007 | Consecutive patients with severe CO intoxication found unconscious who underwent hyperbaric oxygen therapy and in whom MR images were performed at least once within 15 days of exposure | Retrospective cross-sectional study | 16 | 46.6 | MRI | 6/16 developed delayed encephalopathy and all had bilateral diffuse high intensity in white matter of centrum semiovale after relapse of neuropsychicatric symptoms. In 4/6 the same findings had been identified on MRI earlier during a lucid interval. None of the non-delayed encephalopathy group (10/16) had WMH. Basal ganglia T2 hyperintensities were seen in delayed encephalopathy group (3/6) and in non-delayed encephalopathy group (2/10). DWI was acquired in 10/16 (5 in each clinical group): 4/5 of delayed encephalopathy group had DWI positive lesions, n=3 of these had not had any DWI positive lesions earlier during the lucid interval, no DWI lesions in the non-encephalopathy group (0/5) | Not clear | max 15 days | 38% (6/16) |
| Parkinson 2002 | Carbon monoxide poisoned patients with COHb level ≥10% referred from emergency department, consecutive, plus age and sex-matched normal control subjects selected from a normative database. CO exposure = automobile exhaust, combustion engine exhaust faulty furnaces, fumes from charcoal briquettes and from fire | Longitudinal cohort study | 73 | 34.7 | MRI | The CO-poisoned patients (n=9; 12%) had more WMH (PVWMH and CSWMH) vs controls (n=5; 7%). CO subjects had higher PVWMH ratings on day 1 and on day 2 vs controls (t[1,64]= 2.05, p=0.04). Five CO-poisoned patients and one control subject had PVWMH on day 1. | Mean COHb level=22.0 +/- 10.6%. Mean CO exposure duration =22 +/- 70 hours. | MRI acquired within 24-36 hrs, repeated 2 weeks and 6-months | 12% (9/73) |
| Pavese 1999 | Patients referred to hyperbaric oxygen therapy specialist centre for CO intoxication: 22 with acute exposure (majority domestic heating system failures), 8 chronic exposure due to domestic heating system failures x 22 days | Longitudinal observational study | 22 | 39 | MRI | 11/22 had WMH in the acute CO exposure group | Acute mean 1.4 hrs vs 5 hours; chronic mean 22 days (range 12-30 days) | One month (first scan) and 1 year (follow-up scan) | 50% (11/22) |
| Silver 1996 | Patients with acute CO poisoning admitted for hyperbaric oxygen therapy who had had cerebral imaging performed because of unconsciousness on admission and failure or delayed improvement in neurological status after O2 therapy | Cross-sectional observational study | 19 | 33.2 | Both mostly CT | N=7 had bilateral low attenuation areas within the globus pallidus and n=6 had low attenuation changes within cerebral white matter. WML were found in n=3 as an isolated finding and in association with globus pallidus changes in n=2. The white matter changes included focal low attenuation within the centrum semiovale, brain stem, and parietal lobe. | Not clear but all acute | Within 72hrs for 18 CT patients; within 9 days in MRI patients | 37% (7/19) |
| Tom 1996 | Discharge diagnosis of CO toxicity; accidental or intentional CO poisoning | Retrospective record review | 18 | 35.6 | Both mostly CT | Low-density lesions in the globus pallidus (7/18; 39%) and deep white matter changes (5/18; 28%). N=6 CT scans showed no acute changes. ≥1 acute CT finding in 5/18 (28%). | Not clear but all acute | within 1 week of exposure | 44% (8/18) |
| Uchino 1994 | Victims of coal mine explosion 25 years previously, all admitted to hospital unconscious at time of CO exposure. All now have permanent neurological deficits. | Cross-sectional observational study | 13 | 60 | MRI | Bilateral globus pallidus lesions in n=12, degeneration of the white matter in n=12, with focal cortical atrophy. The temporal, parietal, and occipital lobes were usually affected. 7/12 had asymmetrical cortical and subcortical lesions. | Coalmine explosion | 25 years | 92% (12/13) |
| Vieregge 1989 | Patients with coma and midbrain syndrome due to acute CO poisoning given normobaric oxygen therapy. Diagnosis of CO poisoning proved either circumstantially or by COHb determination. Exposure to defective heating equipment, automobile exhaust fumes. | Longitudinal observational study | 4 | 35.5 | CT/MRI (MRI in n=3) | n=4. Individual findings for each case: Patient 1. a Axial CT 4 days after CO poisoning: bi lateral low-density areas in the globus pallidus, b CT scan 24 days  after CO poisoning: slight enlargement of ventricles and cortical sulci.  Low-density areas in the globus pallidus no longer visible, c-e MRI 18  months after CO poisoning: (c) show a small, hypointense lesion in the globus pallidus (d) with small bilateral hyperintense pallidal lesions corresponding to cicatricial cysts. Here and in the neighbouring section (e) a dot-like hyperintense lesion in the frontal white matter is seen.  Patient 2. No data reported - not included  Patient 3. a Axial CT about 10 h after CO poisoning: bilateral low-density areas in the globus pallidus, b CT scan 8 months  after CO poisoning: bilateral low-density areas of the globus pallidus  are still present with slight enlargement of ventricles and cortical sulci. c MRI 18 months after CO poisoning: unilateral hyperintense lesion between the left internal capsule and globus pallidus.  Patient 4. A Axial CT scan about 8 h after CO poisoning: bilateral low-density areas in the globus pallidus, b CT 24 days after  CO poisoning: no pallidal lesions visible  Patient 5. a Axial CT scan 14 days after CO poisoning: bilateral low-density areas in the globus pallidus, b CT 24 days after  CO poisoning: conspicuous low-density changes especially in the subcortical white matter. | CO levels 22, 25,25, 0 (n=4) | CT or MRI within hours-days; for n=3 with follow up MRI/CT< occurred from 8-18months | 100% (4/4) |
| Xiao 2017 | Delayed encephalopathy after acute carbon monoxide poisoning | Unclear | 84 | 42.1 | MRI | in the group administered Ginkgo biloba extract, the WM and globus pallidus lesions of 14 days after treatment were smaller than those in the routine treatment group (CO poisoning care with hyperbaric oxygen) | Unclear | Unclear | 100% (84/84) |
| Yu 2002 | Patients with delayed encephalopathy after acute CO poisoning. All had coma lasting 2-48 hours at onset and all had recovered consciousness | Cross-sectional observational study | 198 | 45.7 | Both  CT in 190; MRI in 59 | 15.2% patients had bilateral basal ganglia lesions, 133 cases (70.0%) had subcortical or centrum semiovale WML on both sides of the frontal lobes, 12.6% with lesions of two types above mentioned and n=4 (2.1%) with no lesions. | Not clear but all acute exposure | Not clear | 67% (133/198) |

## 1B: Carbon disulphide

| **Study**  **(Year)** | **Population** | **Study design** | ***n*** | **Mean age** | **MRI vs CT** | **Imaging abnormalities and location** | **Exposed duration/concentration** | **Exposure-imaging interval** | **SVD % (n)** |
| --- | --- | --- | --- | --- | --- | --- | --- | --- | --- |
| Cha 2002 | Previously employees of a viscose rayon factory, and in whom carbon disulfide poisoning had been diagnosed on the basis of the criteria of the Korean Ministry of Labor | Cross-sectional observational study | 91 | 53.3 | MRI | In 70/91 (76.9%), T2-weighted imaging showed WMH. Lesions detected in 8/9 (88.9%) aged 30-39, 11/15 (73.3%) aged 40-49, 36/47 (76.6%) aged 50- 59, 12/17 (70.6%) aged 60-69, and 3/3 (100%) aged 70-79. 51/91 were in the low exposure group and 40/91 in the high exposure group. The prevalence of WML in these two groups was 76.5% and 75%. Frequent WMH locations were near the frontal horn of the lateral ventricle; corona radiata and centrum semiovale. Lacunar infarcts in 27/91 (29.7%) of Lacunar infarcts in 0/9(0%) aged 30-39, 2/15 (13.3%) aged40-49, 15/47 (31.9%) aged 50-59, 8/17 (47.1%) aged 60-69, and 2/3 (66.7%) aged 70 79. Of 101 lacunar infarcts, 40 (39.6%) were in the basal ganglia, 26 (25.7%) in frontal white matter, ten (9.9%) in the thalamus, nine (8.9%) in the corpus callosum, seven (6.9%) in the pons, five (5.0%) in parietal white matter and four (4.0%) in the external capsule. In the low and high exposure groups, the prevalence of lacunar infarcts was 33.3% and 25.0%, respectively. When compared with previous studies of normal subjects, WMH prevalence was higher among all age groups, especially the younger groups. | 3-27 (mean, 12.9) years | Not clear but previously employed | 77% (70/91) |
| Cho 2002 | Subset of individuals who had worked at a rayon viscose factory in South Korea and were diagnosed with carbon disulphide poisoning by the standard criteria and receiving healthcare services at the local hospital who had undergone MRI | Retrospective MRI review | 311 | 55.9 | MRI | MRI findings revealed a significantly larger number of cerebral lacunae (n=5/12) in the high exposure group. Periventricular hyperintensities mostly located in frontal and occipital areas, and white-matter hyperintensities were mostly in frontal and parietal areas. Cerebral lacunae were more prevalent in the high-exposure group (5/12) vs low-exposure group (1/19), p < 0.022. No significant differences between the two groups in periventricular and whitematter hyperintensities. In multivariate analysis adjusted for age, exposure, and vascular risk factors, lacunae were significantly associated with high carbon disulphide exposure (p < 0.036). No evidence of increasing prevalence of periventricular or white-matter hyperintensities with age. | Mean cumulative exposures were 1069.74 month-ppm in high-exposure group; 198.48 in low-exposure group | mean 10 (5.74) years | 71% (22/31) |
| Huang 1996 | Viscose rayon factory workers (9 cuttermen and 1 spinner) who incurred the most intense and prolonged exposure to CS2, selected from previously studied group of patients who had polyneuropathy after longterm CS2 exposure | Cross-sectional observational study | 9 | 44.5 | Both | Patient 1. Bilateral basal ganglia and corona radiata lesions with cortical atrophy (MRI). Patient 2. Left internal capsule and bilateral corona radiata lesions (MRI) Patient 3. Bilateral basal ganglia and corona radiate lesions (MRI). Patient 4. Bilateral internal capsule lesions (MRI). Patient 5. Normal MRI Patient 6. Mild ventricular dilatation and cortical atrophy (MRI). Patient 7. Normal MRI. Patient 8. No imaging available, not included in analysis. Patient 9. Mild cortical atrophy. Patient 10. Mild cortical atrophy | Estimated 8-hr average in fibre-cutting area: 40-67ppm. Exposure duration:4-23 yrs; mean 15.7 yrs |  | 44% (4/9) |
| Nishiwaki 2004 | Viscose rayon factory workers across 11 factories in Japan (exposed group) and referent workers | Longitudinal case-control study | 666 | 35.9 | MRI longitudinal in 666 | 217 exposed, 125 ex-exposed, and 324 referent subjects: Exposed subjects showed a significantly higher risk for an increase in the number of hyperintense spots on T2-weight images over six years. Odds ratios adjusted for possible confounders in the exposed and ex-exposed workers were 2.27 (95% CI 1.37-3.76) and 1.33 (95% CI 0.70-2.54), respectively. After adjusting for age, in which the dependent variable was ‘‘increased’’ or ‘‘unchanged’’ in number of HIS over six years, OR=2.56 (95% CI 1.59-4.10) in the exposed group and 1.24 (95% CI 0.68-2.27) in the ex-exposed group. The multivariate adjusted odds ratio in the exposed group (adjusted OR 2.27, 95% CI 1.37-3.76) remained significantly higher compared to the referent group. Age was a significant risk factor for an increase in number of HIS, separate from exposure to CS2. | Mean (atmospheric, ppm) and TTCA (urinary, mg/g creatinine) concentrations for past 6 years were 4.9 and 1.6 for all exposed workerss.Mean exposure duration was 19.6 years | 251 remained exposed to CS2 until end of the observation period (exposed workers), and n=140 had their exposure truncated because 4 factories discontinued productio (ex-exposed). Mean period from cessation of exposure to the follow up survey in the ex-exposed subjects was 4 yrs (range 0.8–6.9 yrs) | n/a |

## 1C: Contact sports

| **Study**  **(Year)** | **Population** | **Study design** | ***n*** | **Mean age** | **MRI vs CT** | **Imaging abnormalities and location** | **Duration and concentration of exposure** | **Exposure-imaging interval** | **SVD % (n)** |
| --- | --- | --- | --- | --- | --- | --- | --- | --- | --- |
| Lee 2017 | Professional fighters | Cross-sectional case-control | 561 | 30 | MRI | Microbleed prevalence: 21/498 (4.2%) fighters vs 0/62 (0%) controls, p=0.152). WMC prevalence: 118/499 (23.6%) fighters and 12/62 (19.4%) controls (p=0.525). Total 59 microbleeds: n=1 had 14, n=1 had 10, and the remaining 35 were found n=19 (mean 1.5/fighter). Most microbleeds were located near the cortex, with only a few in the deep white matter and periventricular margins. In the posterior fossa, most cases of CMH were in the inferior aspects of the cerebellum. Most WMC were located near the cortex, and only 2 were located in the periventricular margins. Fighters with WMC trended toward having a higher average of total fights (60 fights in WMH group vs 46 in no WMH group, p =0.098. Fighters with microbleeds had a higher average of total fights than those without (85 versus 47 fights, p=0.530) | Unclear | Not clear but some working and some retired | 24% (118/499) |
| Strain 2013 | Retired professional footballers | Cross-sectional case-control | 26 | 61.8 | MRI | Total and deep but not periventricular WML volumes were significantly different between players with cognitive deficits (n=10) and age-matched controls (n = 20). | Unclear | Unclear | Unclear |
| Zivadinov 2018 | Contact sport athletes and non-contact athlete controls | Cross sectional case-control | 42 | Not reported | MRI 3T | No difference in presence, number, or volume of focal white matter signal abnormalities or in number and volume of microbleeds. 12/21 (57.1%) of contact sport athletes and 11/21 (52.4%) vs controls presented with white matter signal abnormalities. More controls (7/21, 33%) vs contact sport athletes (2/21, 9.5%) had ≥1microbleed (p=.067). Microbleed number (0.6 vs 0.3, 95% CI [−0.5 to – 0.9], p=0.542) and volume (11.2mm3 vs. 2.3mm3, 95% CI [−0.7 to –18.6], p=.077) were also slightly higher in controls | Not clear | not clear but retired from sport | (57%) 12/21 |

## 1D: 1,2-dichloroethane (1,2-DCE)

| **Study**  **(Year)** | **Population** | **Study design** | ***n*** | **Mean age** | **MRI vs CT** | **Imaging abnormalities and location** | **Duration and concentration of exposure** | **Exposure-imaging interval** | **SVD % (n)** |
| --- | --- | --- | --- | --- | --- | --- | --- | --- | --- |
| Chen 2019 | Patients hospitalised with occupational acute severe 1, 2-DCE poisoning working in poorly ventilated private small enterprises | Cross-sectional observational study | 18 | 30.5 | Both | Brain CT (n=18) showed varying degrees of diffuse WML, abnormal signal shadows in bilateral basal ganglia, thalamus, corona radiata and cerebellar dentate nucleus. Brain MRI (n=5/18) showed extensive involvement of white matter in both cerebral hemispheres-abnormal signals in bilateral cerebral white matter, and the involved areas of bilateral cerebral hemisphere subcortical white matter, external capsule, basal ganglia, thalamus and cerebellar hemisphere dentate nucleus were low signal on T1WI and high signal on T2WI T2-Flair | Average working length of (22.45±5.38) months | Not clear but imaging done in acute period | 28% (5/18) |
| Lai 2011 | Patients with 1,2-DCE Chronic Toxic Encephalopathy | Cross-sectional observational study | 10 | 41.5 | MRI | 10 cases show varying degrees of abnormal signal of white matter, low signal intensity on T1WI, high signal intensity on T2WI and FLAIR. MRI could also show extensive abnormal signal in cerebral white matter although the toxic manifestation is mild to moderate. | Unclear | Unclear | 100% (10/10) |
| Liu 2019 | Patients with toxic encephalopathy induced by 1,2-DCE who subsequently had seizures or symptoms of intracranial hypertension, all occupational exposures to 1,2-DCE | Cross-sectional observational study | 7 | 33 | Both: 5 had CT 5 had MRI | CT findings: All lesions appeared as low density and bilateral symmetry. The lesions appeared in white matter of cerebral hemisphere diffusely- CT showed low-density abnormalities of bilateral cerebral hemisphere in all patients including 5 with abnormalities in deep white matter, 3 with abnormalities in thalamus, 4 with abnormalities in globus pallidus, 4 with abnormalities in cerebellar dentate nucleus. MRI findings: All lesions showed high signal intensity on T2WI. All patients had bilateral lesions, among which the most common one was white matter, including: extensive bilateral white matter (n=4), bilateral thalamus (n=3), and globus pallidus (n=4), bilateral cerebellar dentate nucleus (n=4). Both the subcortical and the deep white matter were involved. DW MR showed widespread symmetrical hyperintense changes, consistent with restricted diffusion, involving the globus pallidus, the subcortical and the deep white matter in both cerebral hemispheres | 14 days-7 years | 5 days -1 month | 71% (5/7) |
| Yang 2009 | Patients with toxic encephalopathy induced by 1,2-DCE | Cross-sectional observational study | 5 | 31 | MRI | T1WI low signal and T2WI high signal in bilateral hemispheric white matter, cerebellar dentate nucleus and globus pallidus | Unclear | Unclear | 100% (5/5) |

## 1E: Diving

| **Study**  **(Year)** | **Population** | **Study design** | ***n*** | **Mean age** | **MRI vs CT** | **Imaging abnormalities and location** | **Duration and concentration of exposure** | **Exposure-imaging interval** | **SVD % (n)** |
| --- | --- | --- | --- | --- | --- | --- | --- | --- | --- |
| Hutzelmann 2000 | Compressed air divers with uneventful diving history: 27 commercial and 32 navy divers without history of neurological decompression illness, 48 controls matched for age/BMI/alcohol/smoking | Cross sectional case-controll study | 107 | 43.6 | MRI | All cerebral lesions supratentorial except one cerebellar lesion, lesions were predominantly locatedin subcorticla and central wite matter of the parietal lobes and in the basal ganglia. No periventricular lesions suggesting a primary process such as disseminated encephalitis or primary demyelinating disorder were found. nfocal white matter hyperintense lesions were obesrved in 22/59 (37.3%) of divers and in 23/48 (47.9%) of controls, non=significant. 18% of the nondivers had more than 3 hyperintense spots in their white matter as opposed to 12% of the divers | Not clear | Not clear | 37% (22/59) |
| Palmer 1992 | postmortem professional divers and 15 airmen controls who died in flying accidents aged 24-39 | Cross-sectional postmortem study | 13 | 26.5 | Post-mortem pathology | Grossly distended, empty vessels (presumably caused by gas bubbles) were found in the brains of 15 out of 22 divers who died from diving accidents. Perivascular lacuna formation was found in cerebral and/or cerebellar white matter in three amateurs and in five professionals. In addition to lacuna formation, hyalinization of vessel walls was present in the brains of three amateurs and five professionals. Necrotic foci in grey matter occurred in seven cases and perivascular vacuolation of white matter occurred in seven cases. In one professional diver, there was also unilateral necrosis of the head of the caudate nucleus. | 4-20+ (range) years of professional diving to 35-100 metres | All but one professional died in diving accidents and necropsies were usually carried out within 48h of death, in csae of diving accidents delay was longer | 36% (8/22) |
| Rinck 1991 | Professional male divers with at least one year of professional diving experience; controls= policemen and offshore workers | Cross sectional case-control study | 117 | 38 | MRI | Spots of high signal intensity in white matter on proton density- and/or T2-weighted spin-echo images were detected in 34% of divers vs 42% controls and. The prevalence of changes in divers was inversely related to diving depth, amount of diving, participation in "unsafe diving," and decompression sickness. | N=11 had dived for 1-5yrs, n=29 for 6-10 yrs, n=30 for >10years | At least 48hrs after last dive | 34% (24/70) |
| Sipinen 1999 | Experienced naval divers with similar diving exposures =control group only assessed here | Cross sectional observational study | 29 | 44.8 | MRI | All had normal brain MRI | Not clear but very experienced naval divers | Not clear | 0% (0/21) |

## 1F: High altitude

| **Study**  **(Year)** | **Population** | **Study design** | ***n*** | **Mean age** | **MRI vs CT** | **Imaging abnormalities and location** | **Duration and concentration of exposure** | **Exposure-imaging interval** | **SVD % (n)** |
| --- | --- | --- | --- | --- | --- | --- | --- | --- | --- |
| Alperin 2017 | Astronauts -10 flew long duration mission on the International Space Station and 7 flew a short-duration mission on the Space Shuttle | Longitudinal observational study | 17 | 46.8 | MRI | Significant preflight to postflight changes were measured only in the long-duration cohort and included only the periventricular WMH and ventricular CSF volumes. Changes in deep WMH and brain tissue volumes were not significant in either cohort. The increase in periventricular WMH volume was significantly associated with an increase in ventricular CSF volume (rho = 0.63, p = 0.008). A partial reversal of these increases was observed in the long-duration subcohort with a 1-month follow-up scan. Long-duration exposure to microgravity is associated with an increase in periventricular WMH in response to an increase in ventricular CSF volume in astronauts. At baseline, no significant differences in these measures were found between the 2 cohorts. Preflight to postflight changes in PWMH and ventricular CSF volumes were significant only in the long-duration astronauts, with average increases of 0.21 mL (39%) and 3.1 mL (17%), respectively. No significant differences were observed between the 2 cohorts in deep WMH volumes. In the long duration subcohort, the 1-month follow-up scan demonstrated partial reversal of the increases in PWMH and ventricular CSF volume, with average reductions of 65% and 36%, respectively | Short-duration mission on the ISS Space Shuttle lasting 14 (1.4) days. | Preflight MRI scan was performed ~1 year before departure, and the postflight scan occurred mean 6.1 days after return to Earth. Five of the 10 long-duration astronauts had a second postflight MRI scan 1 month after the first postflight scan | n/a no extractable prevalence data |
| Barisano 2022 | astronauts | Longitudinal observational study | 37 | 48.1 | MRI | MRI performed within days of return detected increased volumes of post-flight PVS (pre-flight white matter PVS mean in NASA space station group 1152mm (SD 559) vs post-flight 1435mm (SD 698), p<0.001; significant findings also detected in basal ganglia PVS; no change detected in controls). | Spaceflight lasting six months | brain MRI scans acquired before and within 2 wk (mean 4.8 [SD 2.2] days) after long-duration spaceflight on the ISS (∼180 d) | Not reported |
| Fueredi 1991 | compressed-air tunnel workers | Cross-sectional case-control study | 30 | 55 | MRI | The 19 subjects in the experimental group had a statistically higher number (p = .05) of white matter lesions (more than 152) than the control group (22 lesions), and 37% of the experimental group had more than 20 white matter lesions each (seven of 19 subjects) while only 18% of the control group had 10 or 11 lesions each. The experimental group had a five times higher risk vs control group of having highgrade lesions, and a high statistical correlation (p = .02) was found between the number and severity of lesions in the experimental vs control group when linear trend analysis was performed. The distribution of white matter lesions was 100% in the centrum semiovale and 50% in the optic radiations. Additionally, three subjects in the experimental group had two internal capsule lesions and one basal ganglia lesion. An odds ratio was also calculated, which showed a fivefold increase in the likelihood of an exposed individual having a grade 3 or 4 WML compared with an age-matched control subject. The 90% confidence interval for the odds ratio ranged from an increased risk of 1.13 times to 22.2 times. | Not clear | not clear but current workers | 53% (10/19) |
| Hupfeld 2022 | astronauts | Longitudinal observational study | 26 | 47.46 | MRI | Following spaceflight, there was an increase in total PVS volume from pre- to post-flight in novice astronauts (estimate for change in total PVS volume (mm^3^/cm^3^ of WM) = 0.16; [SE 0.06]; p = 0.020) but change in PVS number. Experienced astronauts had decreased PVS volumes postflight. There was an additional trend towards higher pre-flight PVS load and greater flight experience (r=0.60–0.71; p > 0.05). | Spaceflight lasting six months | Mean 4.53 (SD 1.13) days | Not reported |
| Jersey 2013 | Ultra-high altitude (U-2) pilots referred to hospital for evaluation of neurological decompression sickness following hyperbaric oxygen treatment who had MRI performed | retrospective cross-sectional study | 13 | 35.4 | MRI | Two (15%) demonstrated acute subcortical lesions on MRI, seven (54%) had asymptomatic WMHs, and six (46%) were normal. Only one pilot (MP#7) with severe neurological DCS symptoms had acute lesions on initial exam immediately following HBO therapy. These findings have been reported previously and included multiple T2/FLAIR hyperintensities in the cortical and subcortical white matter of the parietal and frontal lobes, the T2 hyperintense abnormalities persisted to a lesser degree on serial MRI exams performed up to 7 mo later. Only one other U-2 pilot (MP#17) demonstrated an acute brain lesion on MRI immediately following (within 12 h) a neurological DCS incident. For both cases (MP#7 and MP#17), lesions occurred in the cortical graywhite matter junction. | median 270.5 flight hours (IQR 93.5 - 879) | not clear but MRI done as part of usual clinical care following acute DCS incident involving CNS at facility | 54% (7/13) |
| Kang 2014 | Military fighter pilots visiting aeromedical centre for checkup | Cross-sectional observational study | 81 | 33 | MRI | Brain MRI showed more than one WML in 36 pilots (44.4%). No PVWMLs in pilots with identified WMLs on brain MRI, whereas DWMLs were shown in all pilots with WMLs identified on brain MRI. All DWMLs were located in the frontal lobes with patterns of punctate DWMLs except for 2 cases with coexisting parietal lobe or temporal lobe lesions. Pilots with WMLs: older, higher fasting blood sugar levels. | mean total flying experience = 954.642 ± 622.824 h | not clear but still working as pilots | 44% (36/81) |
| Lim 2012 | Airforce pilots on active flight duty. Control group = nonflying Republic of Korea Air Force personnel with no flight experience | Cross-sectional case-control study | 62 | 51.1 | MRI | There was no statistical difference between the pilots and non-flying personnel for WMH findings (54.8%/ vs. 32.3%, p=0.073 ). Number of WMH pilots 5.2 ( 9.44) nonflying personnel 4.6 (10.15), p= 0.825. no. of WMH not associated with airraft type,, total flight hours, altitude. Of the factors related to flight, only the flying altitude (OR 1.005, (1.001-1.009)]) was significantly related to the presenec of WMH on multivariate analysis | Mean total flight hours of pilots 3025 hours | not clear but all still working on active flight duty | 55% (17/31) |
| McGuire 2013 | U-2 pilot occupational exposure to hypobaria in active duty members in the US airforce / controls =active-duty military members with a doctorate degree assigned to local duty | Cross-sectional case-control study | 193 | 37 | MRI 3T | U-2 pilots exposed to hypobaria demonstrated an increase in volume (394%; p = 0.004) and number (295%; p < 0.001) of WMH. Analysis of regional distribution demonstrated WMH more uniformly distributed throughout the brain in U-2 pilots compared with mainly frontal distribution in controls. Pilots demonstrated a nearly 4-fold increase in volume (375%) and a 3-fold increase in the number (294%) of WMH; this difference was significant for both the raw data and the site-specific adjusted data. Spearman correlation coefficient between lesion volume and age approached significance for the normal controls but not for the pilots (p 5 0.07 and 0.16; r 5 0.19 and 0.14 for controls and pilots, respectively). The correlation coefficients between the number of lesions and age were not different (p 5 0.14 and 0.2; r 5 0.15 and 0.13 for controls and pilots, respectively). Correlation coefficients between the volume and number of WMH and the number of U-2 flight hours were not different for the pilots (p . 0.5; r 5 0.05 and 0.03 for volume and number, respectively) | N Dear Elaine,  could you please send some dates and then a formal invitation to Yvonne, below? She should be expecting an email from you.  Many thanks,  Unaot clear | not clear but all active duty members | n/a mean WMH volumes rather than prevalence data |
| McGuire 2014 | Eighty-three altitude chamber personnel, active duty members of the US military exposed to nonhypoxic hypobaria (PHY), 105 U-2 pilots (U2P), and 148 age- controlled and healthmatched doctorate degree controls (DOC) | Cross-sectional case-control study | 336 | 37.1 | MRI | Subcortical WMHs were more prevalent in PHY (volume p 5 0.011/count p 5 0.019) and U2P (volume p < 0.001/count p < 0.001) when compared to DOC, whereas PHY were not significantly different than U2P. | All PHY had experienced >50 occupational exposures to >25,000 feet altitude with duration of 30 to 60 minutes. Exposure frequency was variable but not more often than every third day, although occasionally mission demands required every other day exposure. U2P are exposed to hypobaric cabin altitudes (28,000–30,000 feet) for up to 9 hours with a variable frequency, not more often than every third day. | not clear | n/a compares mean WMH volumes |
| McGuire 2019 | N=64 healthy aircrew undergoing standard US Air Force altitude chamber training vs N=60 controls not exposed to hypobaria | Cross-sectional case-control study | 124 | 21 | MRI | No significant changes were observed in structural measurements including manually traced WMH/total WMH volume | 5000ft descent to sea level (exposure 12.2 psi/632 mmHg/ambient air O2 via aviator mask>500 mmHg) followed by 100% O2 at sea level, ascent to 25,000ft (exposure 5.45 psi/282 mmHg/ambient air O2 via aviator mask >200 mmHg), then descent to 18,000ft | 24h and 72h post-exposure | n/a no prevalence or mean volume data available |
| Pimenta 1999 | Occupationally exposed to large pressure amplitude and low pressure noise and referred to ENT and neurology due to severe balance disorders | Cross-sectional observational study | 20 | 35 | MRI | hyperintense foci in T2 of the subcortical white matter in 10/20 and normal in remainder. | symptoms began over 15 years (SD 8) after beginning activities | symptoms began over 15 years (SD 8) after beginning activities | 50% (10/20) |

## 1G: Lead

| **Study**  **(Year)** | **Population** | **Study design** | ***n*** | **Mean age** | **MRI vs CT** | **Imaging abnormalities and location** | **Duration and concentration of exposure** | **Exposure-imaging interval** | **SVD % (n)** |
| --- | --- | --- | --- | --- | --- | --- | --- | --- | --- |
| Bleecker 2007 | lead smelter workers under age 50, currently employed workers at a primary lead smelter, routinely exposed to lead dust and vapor | Cross-sectional observational study | 61 | 40 | MRI | WMC were present in 23% of MRIs. The distribution of WMC grades on MRI was as follows: grade 0 = 77%, grade 1 = 10%, grade 2 = 7%, grade 3 = 5%, grade 4 = 2%. . The group with WMC was significantly older, less educated, and had more years of employment. Risk factors for WMC in this group were higher for history of HTN, current tobacco use, cholesterol, triglycerides, and CRP but only cholesterol was significantly different. WMC was not correlated with age but was significantly negatively correlated with education and positively correlated with PbBn and IBL | Employment duration: 19years (1–26), current blood lead (PbB) was 29 (16–42) mg/dl, working lifetime weighted integrated blood lead (IBL) was 826 (65–1451) mg year/dl, working lifetime weighted average blood lead (TWA) was 42 (17–59) mg/dl, and bone lead (PbBn) was 39 ( 12–90) mg Pb/g bone mineral | not clear but all current employees | 23% (14/61) |
| Schwartz 2010 | former organolead workers from a chemical manufacturing plant , involved in the manufacture of tetraethyl lead + tetramethyl lead | Longitudinal  observational study | 362 | 60.8 | MRI | For the change in CHS white matter lesion grade score (CHS2 minus CHS1), 6 persons (1.7%) improved by one category, 87 persons (24.0%) were unchanged, 134 persons (36.9%) wors ened one category, 100 persons (27.6%) worsened by two catego ries, 24 persons (6.6%) by three categories, and 12 (3.3%) by four or five categories. Neither peak tibia lead nor control status were associated with change in CHS sores. Baseline age and increasing duration between MRIs were associated with increases in CHS scores (beta= 0.055, P =0.001 and beta 0.286, P=0.05, respectively). | mean 8.5 (9.6) years of employment in lead factory | mean 18.0 (10.9) years since last lead exposure at study recruitment (which was approx four years before first MRI) | n/a no extractable prevalence data |

## 1H: Military

| **Study**  **(Year)** | **Population** | **Study design** | ***n*** | **Mean age** | **MRI vs CT** | **Imaging abnormalities and location** | **Duration and concentration of exposure** | **Exposure-imaging interval** | **SVD % (n)** |
| --- | --- | --- | --- | --- | --- | --- | --- | --- | --- |
| Donna-Ferreira 2019 | Community dwelling veterans >64 years with at least two vascular risk factors. | Cross-sectional observational study | 60 | 64 | n/a – VaD vs no VaD | 7/60 (11.6%) had vascular MRI or vascular dementia diagnosis | not clear | not clear but all retired | n/a – VaD vs no VaD |
| Liu 2016 | Clinical diagnosis of TBI in active duty members between 18-60, mostly chronic TBI | Cross-sectional observational study | 603 | 33.8 | MRI | Microbleeds were identified in 43/603 patients. Patients with traumatic brain injury (TBI) showed a reduced number of cerebral microhemorrhages (CMHs) at follow-up compared with baseline images (mean 6 standard deviation, 9.8 microhemorrhages 6 12.8 vs13.7 microhemorrhages 6 16.6; p = .019). n The total volume and mean magnetic susceptibility of the CMHs in patients with TBI both decreased over time: 20.85 mm3 per day 6 1.59 for total volume (P = .039) and 20.10 parts per billion per day 6 0.14 for mean magnetic susceptibility (P=0.016). | unclear | median 856 days | 7% (43/603) |
| Lotan 2018 | military service members with history of chronic blast-related TBI drawn from ongoing study of military veterans | Cross-sectional observational study | 146 | 32.8 | MRI | No cerebral microhemorrhages were identified in any subject | Sixty-nine subjects (47.3%) had 2 episodes of mTBI (in conjunction with close proximity to a blast explosion without concomitant blunt traumatic head injury based on the Department of Veterans Affairs and the Department of Defense definition of mTBI) | 9.4 (SD 6.2) years | 0% (0/146) |
| Lwi 2019 | Female army veterans aged ≥65 obtaining care at the Veterans Health Administration | Cross-sectional observational study | 16728 | Not reported but>65 | n/a – VaD vs no VaD | 23.5% had vascular dementia (Alzheimer's disease was the most prevalent at 72.7%). 16728/168111 total sample had dementia | not clear | not clear | n/a – VaD vs no VaD |
| Martindale 2018 | Veterans with history of blast exposure in Iraq and Afghanistan without a lifetime history of TBI | convenience sample from longitudinal observational study | 19 | 39 | MRI | MRI WMH changes were associated with severity of blast exposure. Although most participants were in the normal range at both Time1 and Time2, the median total number of WMHs and total WMH volume were significantly higher at Time2. This was primarily due Tto four participants (IDs 7, 8, 14, 19). All four participants had blast exposure at Time1, two had TBI at Time1, one of which had another TBI by Time2. Visual comparisons of sectional images from T1 and T2 indicated that the higher quality of imaging at T2 was an influence. At Time1, the severity of blast exposure was significantly correlated with WMH number (r = .72, p = .011) and there was a trend towards WMH volume (r = .73, p = .061). Number of blasts was not correlated with either WMH number (r = –.06, p = .851) or volume (r = –.16, p = .735) at Time1. At Time2 severity of blast exposure was significantly correlated with both number of WMHs (r = .76, p = .007) and WMH volume (r = .69, p = .019). Number of blasts was not correlated with either number of WMHs (r = –.06, p = .86) or WMH volume (r = –.14, p = .678) at T2. Regarding change between Time1 and Time2, there were significant associations between severity of blast exposure on changes in WMH number (r = .54, p = .018) and volume (r = .50, p = .031). There was no association between number of blasts on change in WMH number (r = –.07, p = .769) or volume (r = –.09, p = .720). There was also no association between new TBI and change in WMH number (r = .21, p = .384) or volume (r = .27, p = .257). disc - , we detected significant increases in WMH number and volume, such that a greater number and volume of WMHs were seen at T2 compared to T1. This was primarily due to changes in 4/11 who completed imaging, all with blast exposure at T1. However, five other participants also had blast exposure at T1 without significant increase in WMHs at T2. | average 1-4 combat deployments | less than 9 years (2001-2010) | n/a correlation data |
| Patel 2020 | Cases=military personnel with ≥3 persistent symptoms from active duty mild TBI 3 months-5 yrs before enrollment. Cases are from BIMA study, a RCT of hyperbaric oxygen in US military personnel with persistent postconcussive symptoms after mTBI | Cross-sectional case-control study; cases from RCT and controls from observational study | 146 | 33 | MRI | The odds of >=1 WM hyperintensity in the brain-injured group was about 3.5 times the odds for healthy controls (95% CI, 1.58-7.72; P = .002) after adjustment for age. Similar findings were seen for frontal lobe WMH (OR = 2.43; 95% CI, 1.16–5.08; P = .02). Median total WMHs in the mTBI and healthy controls groups were 3 (range, 0–108) and 1 (range, 0–83), respectively. Increasing white matter lesion burden in the mTBI-versus-healthy controls cohort was not observed in other regions | not clear | cases: time since injury ranged from 4 to 60 months (mean, 25.6 6 16.2 months), | n/a OR |
| Piantino 2021 | veterans recruited from VA Puget Sound parent study, included if MRI data of sufficient quality for PVS segmentation plus history of mTBI due to blast or any other cause | Cross-sectional observational study | 56 | 32 | MRI | significant positive relationship between the number of mTBIs sustained in the military and both PVS number and volume (p = 0.04) | not clear | Months between mTBI and MRI=median (IQR) 55 (40–95) | n/a nil extractable |
| Riedy 2016 | military service members with clinical diagnosis of TBI - Participants who did not have TBI (n = 42) consisted of active-duty service members or dependents with no diagnosis of TBI | Cross-sectional case control study | 876 | 34 | MRI | The most common finding in our primarily mild chronic TBI population was the presence of dilated perivascular spaces (64.3% [536 of 834] vs controls 57.1% of n=42); OR, 1.35 [95% CI: 0.72, 2.53], p=0.22) and T2-weighted hyperintense areas in the white matter (51.8% [432 of 834] vs controls 38.1% of n=16/42; OR, 1.75 [95% confidence interval: 0.92, 3.30], p=0.57). Presumed TBI-related findings, such as microhemorrhage, 6.64 [95% confidence interval: 0.40, 109.22]) of TBI participants were noted in 7.2% (60 of 834; OR, and showed increased incidence with TBI severity (P < .001, moderate and severe vs mild). T2-weighted hyperintense areas and microhemorrhages did not collocate by visual inspection..Postcontrast T2-weighted FLAIR enhancement was observed in 13.7% (114 of 834 vs 2.4% of n=42; OR, 6.49 [95% confidence interval: 0.88, 47.66], p=0.018) of participants with TBI, and surface veins were the most likely source. The comparison population without TBI had no evidence of microhemorrhage. | TBI severity defined according to DDVA criteria. Of 817 TBI, blast-related TBI incidence was 84.2% (688); 68.7% (561) experienced multiple blasts, and 25.9% (212) reported two or more blasts or injuries within 1 month. | mean 1381 days (SD 1489) | 51% (432/834) |
| Tate 2017 | active military service members | Cross-sectional observational study | 152 | 34.9 | MRI | FLAIR white matter hyperintensities (WMHs) were present in all three groups at statistically similar rates (41% mTBI, 49% OI, and 29% PTSD). With the exception of a single OI participant showing a small discrete SWI lesion, SWI abnormalities were overwhelmingly present in mTBI patients (22% mTBI, 1% OI, and 0% PTSD) | not clear | not clear | 0% (0/152) |
| Wang 2022 | 55 Vietnam War veterans with a history of TBI and 52 non-TBI Vietnam War veterans from ADNI | Cross-sectional observational study | 107 | 68.5 | MRI | Mean WMH volume=5.8mL (SD 4.6) in military control and WMH vol 5.0 (7.4) in TBI military. Severe pWMH in 20 (38.5%) of military controls and in 13 (23.6%) of TBI military. Severe dWMH in 14 (26.9%) of military controls and in 12 (21.8%) of TBI military. CSO PVS number 14.1 (8.4) in military controls vs 20.3 (9.7) in TBI military. BG VPS number 7.7 (3.8) in military controls vs 8.0 (4.4) in TBI military | Not clear | Not clear but all were veterans, participating in ADNI study | Not reported |

## 1I: Pesticides/fertilisers

| **Study**  **(Year)** | **Population** | **Study design** | ***n*** | **Mean age** | **MRI vs CT** | **Imaging abnormalities and location** | **Duration and concentration of exposure** | **Exposure-imaging interval** | **SVD prevalence** |
| --- | --- | --- | --- | --- | --- | --- | --- | --- | --- |
| Hebert 2000 | population-based cohort study | cohort incidence study following individuals free of dementia x 5 years | 8623 | 69.4 | n/a – VaD vs no VaD | Risk factors for incident VaD included occupational exposure to pesticides or fertilizers (2.05) (1.03 - 3.85) but not plastic or rubbers 1.75 (0.57 to 4.45) | not clear | not clear but all >65/retired | n/a OR for vascular dementia |

## 1J: Solvents

| **Study**  **(Year)** | **Population** | **Study design** | ***n*** | **Mean age** | **MRI vs CT** | **Imaging abnormalities and location** | **Exposure duration and concentration** | **Exposure-imaging interval** | **SVD % (n)** |
| --- | --- | --- | --- | --- | --- | --- | --- | --- | --- |
| DelBigio 2009 | postmortem study solvent inhalation: 28 died of acute intoxication, 21 hanging, 8 trauma, 9 sepsis/aspiration, 4 smoke inhalation/burns in fire, 4 hypothermia | Retrospective post mortem study | 88 | 37 | n/a – post-mortem pathological study | 16/88 cases had well-established leukoencephalopathy and perivascular space enlargement with multifocal perivascular myelin loss and inclusion-containing macrophages. Six/88 cases (age 15–55, median 27 years) had early leukoencephalopathy with scattered macrophages but no obvious myelin changes. | not clear | variable but organic chemicals, including toluene, were measured in the blood of 52/88 cases, suggesting acute exposure prior to death | 25% (22/88) |
| Keski-Santti 2009 | Chronic Solvent Encephalopathy patients who had MRI not CT | Cross-sectional observational | 71 | 51 | MRI | Abnormal WMH were found in 20/71 (28%). Mostly located both in the watershed and other WM areas; in n=3 located outside watershed.10/14 with small focal WMH also had large focal WMH. N=2 also had focal confluent WMH and n=1 had diffuse confluent WMH. Periventricular and brainstem WMH were related to age. | mean 10.8 (4.4) Occupational Exposure Limit Years | not clear | 28% (20/71) |
| Thuomas 1996 | N=32 exposed to industrial solvents daily for 5-28 yrs (t) with CNS symptoms vs 40 age-matched, healthy controls | Cross-sectional case-control study | 72 | 43 | MRI | Exposure to thinner, trichloroethylene, xylene, and white spirit.All patients showed decreased signal in the basal ganglia on T2-weighted images. In n=11, the white matter showed diffuse hyperintensity with loss of the grey-white matter discrimination and with distinct periventricular hyperintensities in n=5. The controls had no pathological changes in the brain. -- All patients showed decreased signal in the putamina; 8 of these patients also had decreased signal in the caudate nuclei and 12 patients in the thalami. N=3 had decreased signal intensity in the caudate nuclei and in the thalami. The reference group had no pathological changes in the basal ganglia or the white matter, nor any cortical atrophy. | 5-28 years | not clear | 100% (32/32) |
| Yamanouchi 1995 | Chronic solvent abusers for at least one year mostly laquer thinner containing toluene | Cross-sectional observational study | 20 | 21.8 | MRI 1.5T | WMH in cerebrum, brain stem, and cerebellum on T2-weighted images in n=7/20. The patients with diffuse white matter change showed obvious brain atrophy, including hippocampal atrophy and thinning of the corpus callosum. According to the hyperintensities on PD–weighted images, white matter change was characterized as follows: (a) restricted white matter change (ie, white matter change restricted to periventricular portion, and the demarcation between gray and white matter was preserved; (b) diffuse white matter change (ie, white matter change more widespread, and the demarcation between gray and white matter was lost); and (c) intermediate white matter change (ie, demarcation between gray and white matter lost in some portions). The patients with diffuse white matter change mainly abused lacquer thinner for longer periods than did the patients with restricted white matter change and intermediate white matter change, who mainly abused pure toluene. | mean exposure 6.7 (SD 2.1) years | mean 7.2 (4.0) years of abuse (range 1 to 16). Most abstained from solvents at least 3 weeks before MRI examination | 35% (7/20) |

## 1K: Toluene

| **Study**  **(Year)** | **Population** | **Study design** | ***n*** | **Mean age** | **MRI vs CT** | **Imaging abnormalities and location** | **Duration and concentration of exposure** | **Exposure-imaging interval** | **SVD % (n)** |
| --- | --- | --- | --- | --- | --- | --- | --- | --- | --- |
| Aydin 2002 | homeless patients admitted for a solvent-abuse rehabilitation program who chronically abused liquid paint thinner | Cross-sectional observational study | 41 | 17.5 | MRI | WML in 46% of the patients (periventricular white matter and the centrum semiovale-19 (46%), cerebellar white matter 15 (37%), internal capsule 13 (32%), brain stem. Thalamic hypointensity in 8(20%). WMC were restricted in 53% and diffuse in 47%. The development of WMC and thalamic hypointensity were signficantly associated with duration of abuse longer than 4 years (P< .05 and P< .01, respectively). Patients with diffuse white matter changes had abused the substance longer than had patients with restricted changes. | Mean duration of abuse was 4.6 years (range, 1–11 years), daily quantity consumed highly variable | 4-7 days of abstinence between last abuse and MRI | 46% (19/41) |
| Caldemeyer 1996 | chronic toluene abusers | Retrospective cross-sectional study | 6 | 35 | MRI | All had abnormal areas of increased signal intensity in the white matter, degree and extent were highly variable. N=3 had minimal focal white matter abnormality involving the subcortical white matter (n = l) and corpus callosum (n = 2. N=3 showed more extensive T2 hyperintensity involving the corticospinal tract (n = 2), pons (n = 3), cerebral peduncles (n = 2), cerebellar hemisphere (n = l), and capsular (n = 3), subcortical (n = 2), and periventricular (n = 3) white matter. Patient 1, who had the shortest duration of toluene abuse, had the most extensive T2 hyperintensity that not only involved the white matter diffusely, but also involved the basal ganglia and thalami. Patient 1 had a unique ringlike lesion involving the left internal capsule, globus pallidus, and putamen. There was no correlation between the severity of white matter changes on MR images and the presence of T2 hypointensity or duration of toluene abuse. Patient 5 had a follow-up examination months after the initial examination that showed progression of the existing white matter disease and development of new lesions in the medulla, corpus callosum, and brachium pontis. He had continued to abuse toluene. Patient 1 MRI one-month later showed decrease in lesion size and loss of rim and progression of the T2 hyperintensity. In 5/6 (83%), there were areas of abnormal hypointensity on T2-weighted images. Four showed abnormal hypointensity of the thalami. Other areas of involvement included the globus pallidus (n = 3), putamen (n = 2), caudate (n = 2), substantia nigra (n = 5), red nucleus (n = 4), pons (n = 4), anterior commissure (n = 4), medulla (n = 1), brachium pontis (n = l), and cerebellar hemispheres (n = 1). | chronic toluene abuse ranging from 5 months to 15 years | 5/6 ongoing exposure, 1/6 last exposed 10 years ago | 100% (6/6) |
| Rosenberg 1988 | Chronic toluene abusers | Cross-sectional observational study | 11 | 30 | MRI | 3/11 had abnormal scans: increased periventricular signal intensity was seen in all, and was severe and extensive in n=2 | Mean 117 months; average consumption two cans (25 1/2 oz) each day (range 1-3 cans) ~ 350mg toluene daily | 9/11 had abstained x 1 month | 27% (3/11) |
| Rosenberg 1988 | Chronic toluene abuse for ≥7years, all attending a toxic vapour rehabilitation program | Cross-sectional observational study | 6 | 30 | MRI | MRI in 6 chronic toluene abusers revealed increased periventricular white matter signal intensity on T2-weighted images. | Mean duration of abuse=168 months (SD 46). Average consumption was two cans (=25oz) each day (range, 1-3 cans),~350 mg toluene daily. Aerosol products contained 59-61% toluene, 10% methylene chloride and traces of xylene | not clear but all in rehab program | 100% (6/6) |

## 1L: Other occupational categories

| **Study**  **(Year)** | **Population** | **Study design** | ***n*** | **Mean age** | **MRI vs CT** | **Findings** | **Duration and concentration of exposure** | **Exposure-diagnosis interval** |
| --- | --- | --- | --- | --- | --- | --- | --- | --- |
| GarciaGarcia 2001 | >65 population-based study including community-dwelling and institutionalised patients | Cross-sectional observational study | 3214 | 74.3 | n/a – VaD vs no VaD | prevalence of VaD was 1.8% of population. vascular dementia and blue collar workers OR 2.23, 0.56-10.5 houseworkers 1.68 (0.29-9.65) farmers 0.45 (0.07-2.74) | not clear | not clear but all >65yrs |
| Gracia-Rebled 2022 | Population based study assessed for incident vascular dementia | Cross-sectional observational study | 3883 | 72 | n/a – VaD vs no VaD | Male agricultural workers had the highest risk of VaD (HR 1.45;  95% CI 0.41-5.18), although this was non-significant. After adjusting for vascular RFs, this association is completely lost (HR 0.72; 95% CI  0.18-2.83) Females: VaD risk 2x in blue-collar workers and 2.7x in those whose main occupation was housework compared to those in white collar jobs, but not statistically significant in the multivariate model. | not clear | Not clear but mean age =72 and refers to main occupation during lives |
| Helmer 2001 | population-based cohort study, community dwelling over 65s | Cross-sectional observational study | 2950 | Not reported but all >65 | n/a – VaD vs no VaD | Farmers had the higher incidence of vascular dementia. VaD: Housewives/inactives (n=11) 0.61 %py (person years); farmers (n=28) 1.02%py; domestic services (n=15) 0.93%py; blue collar workers (n=16) 0.54%py; other employees (n=22) 0.42 %ppy; craftsmen/shopkeepers (n=14) 0.53 %py; professionals/managerials (n=6) 0.31%py; total pop (n=112) 0.59%py developed VaD | main occupation during active life | not clear but all >65yrs |
| Mortel 1995 | sample of >60yr old participants from ongoing prospectigve studies of aging and dementia over 15 years | Cross-sectional observational study | 102 | 71.2 | n/a – VaD vs no VaD | Unadjusted univariate analyses: Farmers 2.4 (0.20-28.21) Domestics incalculable Blue collar 5.22 (2.36-11.54) white collar 2.99 (1.50-5.95) crafsmen/shopkeepers 3.60 (0.73-17.78) homemakers incalculable. Logistic regression models adjutsed for age but did not explicitly assess occupational categories | not clear | not clear but all >60 years |
| Ravaglia 2002 | Older people living in Northern Italy | Cross-sectional observational study | 1016 | 74.6 | n/a – VaD vs no VaD | After adjustment for age and gender, education but not occupation was associated with both AD and VD | not clear | not clear but all >65 |
| Seidler 2007 | Cases and controls ascertained from records of 23 GP practices and based on MMSE and Hachinski Ischaemic Score applied to clincal records. Differential diagnosis based on ICD 10,all medical records reviewed by psychiatrist | Cross-sectional observational study | 59 | 78 | n/a – VaD vs no VaD | Having worked as an electrical and electronics worker (OR for 10 years 3; 95% CI 1.2 to 7.6) as well as a food and beverage processor (OR for having ever worked as a food and beverage processor 7.3; 95% CI 2.0 to 27.3) and a labourer (OR for having ever worked as labourer 6.3; 95% CI 1 to 39.2) was associated with possible vascular dementia. However, the numbers are very small. Blue-collar work in general was not significantly associated with vascular dementia. Cumulative exposure to magnetic fields and maximum exposure to magnetic fields were not significantly associated with any diagnosis of dementia | not clear | median time interval between last job phase and dementia diagnosis was 17 years (men); 24 years (women) |
| vanLoenhoud 2019 | Alzheimer's disease (AD) (n = 1467), frontotemporal (n = 281), VaD (n = 98), Lewy body disease (n = 174), and progressive supranuclear palsy/CBD (n = 101) selected from the Amsterdam Dementia Cohort | Cross-sectional observational study | 2121 | 67.2 | n/a – VaD vs no VaD | Vascular dementia was relatively common in the Transportation/Logistics sector, and higher vascular risk factors partly explained this relationship. The adjusted residuals revealed three effects significant at p < .001. First, individuals from the Transportation/Logistics sector were more often diagnosed with VaD (adjusted residual 4.0). Second, this occupational class had fewer AD participants (adjusted residual − 3.7). uncorrected logistic regression models with either AD or VaD as a dependent variable (AD/VaD = 1 in separate models, other diagnosis = 0) confirmed transportation/Logistics to be significantly related to both VaD (β = 1.23, odds ratio [OR] = 3.41, p < .01) and AD (β = − .85, OR = .43, p < .001). Forward selection of explanatory variables in corrected models revealed that VRF score, but no other factors (i.e., age, sex, education), was positively associated with a VaD diagnosis (β = .43, OR = 1.53, p < .001) and reduced the Transportation/Logistics effect by 15% (from β = 1.23 to 1.04, OR = 3.41 to 2.84). | not clear | not clear but all >40 |
| Whalley 1995 | Dementia patients (AD VaD) admitted to Scottish Mental Hospitals between 1974-88, occupations obtained from death certs or case notes | Retrospective record review | 746 | Not reported | n/a – VaD vs no VaD | Among male AD and VaD cases no single occupation showed any significant difference from the proportion in the general population estimates | unclear | not clear |
| Zhang 2006 | population survey of community residents aged > 55 years | Cross-sectional observational study | 1027 | 43.7% aged 55-64; 39.2% 65-74; 14.4% 75-84; 2.7% 85+ | n/a – VaD vs no VaD | professionals had statistically significant and borderline lower prevalence odds for both VaD and AD; sales-service occupations had significantly lower odds for AD only. Farmer OR (1; 1-1.0); nonfarm laborer 0.6 (0.3-1.1); official 0.2 (0.2-1.2); professional 0.1 (0.03, 0.4); sales service 0.4 (0.1,1.7); housework 0.7 (0.3,1.5). For VaD, all occupations showed lower prevalence odds compared to farmers, but these differences were statistically significant only in one group, professional workers (pOR fully-adj = 0.1, 95% CI 0.03–0.4). | not clear | not clear |

## Supplementary Table 2. Terms used to describe radiological features of SVD

| **Terminology** | **No. of studies using term** |
| --- | --- |
| White matter hyperintensities | 18 |
| White matter lesions | 11 |
| T2 hyperintensities/high signal intensity involving white matter | 10 |
| (CT) Low density lesions in the white matter | 6 |
| Hyperintensity involving subcortical region/white matter | 4 |
| Hyperintense subcortical lesions in white matter on T2-weighted imaging and low intensity on T1 | 5 |
| Focal lesions in white matter | 4 |
| White matter changes | 3 |
| Leukoencephalopathy & EPVS | 1 |
| White matter signal abnormalities | 1 |
| Cerebral microhaemorrhages | 3 |
| Lacunae | 2 |
| Perivascular spaces | 4 |
| Lacunar infarction | 2 |
| Acute subcortical lesions | 1 |
| Basal ganglia, corona radiata, and internal capsule lesions | 2 |
| Acute brain lesions in deep nucleus, cerebellum, white matter | 1 |
| Degeneration of the white matter | 1 |

## Supplementary Table 3. Risk of bias assessment

Red = high risk of bias; yellow = low risk of bias

|  | **Selection of participants** | **Confounding variables** | **Exposure measurement adequate** | **Blinding of outcome assessment** | **Incomplete outcome data** | **Selective outcome reporting** |
| --- | --- | --- | --- | --- | --- | --- |
| **Carbon monoxide** | | | | | | |
| Chang 1992 | low | high | low | high | low | low |
| Chang 2010 | low | high | high | high | low | low |
| Chen 2005 | low | high | high | high | low | low |
| Choi 1993 | high | high | high | high | low | low |
| Durak 2005 | high | high | high | high | low | low |
| Fukuhara 1996 | low | high | high | high | high | high |
| Hao 2017 | low | high | high | high | low | low |
| Hsiao 2004 | low | high | low | high | low | low |
| Kim 1980 | high | high | high | high | low | low |
| Kim 2003 | high | high | high | high | low | low |
| Kim 2017 | low | high | low | high | low | low |
| Lee 1994 | low | high | low | high | low | low |
| Liu 2001 | low | high | high | high | low | low |
| Liu 2020 | low | high | low | high | high | low |
| Matsushita 1996 | low | high | high | high | low | low |
| Mimura 1999 | low | high | low | high | low | low |
| Miura 1985 | low | high | high | high | low | low |
| Nah 2020 | high | high | low | low | low | low |
| O'Donnell 2000 | low | high | low | low | low | low |
| Otubo 2007 | low | high | low | high | low | low |
| Parkinson 2002 | low | high | low | low | high | low |
| Pavese 1999 | low | high | low | high | high | low |
| Ren 2022 | low | high | low | high | low | low |
| Silver 1996 | low | high | low | high | low | low |
| Tom 1996 | low | high | low | low | low | low |
| Uchino 1994 | low | high | low | high | low | low |
| **Other occupations** | | | | | | |
| GarciaGarcia 2001 | low | low | low | low | low | low |
| Gracia-Rebled 2022 | low | low | low | high | low | low |
| Helmer 2001 | low | high | high | high | low | low |
| Mortel 1995 | low | high | low | high | low | low |
| Ravaglia 2002 | low | low | high | high | low | high |
| Seidler 2007 | low | high | high | low | low | low |
| vanLoenhoud 2019 | low | low | high | high | low | low |
| Whalley 1995 | high | high | high | low | high | low |
| Zhang 2006 | low | low | low | high | low | low |
| GarciaGarcia 2001 | low | low | low | low | low | low |
| **Military** | | | | | | |
| Donna-Ferreira 2019 | high | high | high | low | low | low |
| Liu 2016 | low | high | low | high | low | low |
| Lotan 2018 | low | high | low | high | low | low |
| Lwi 2019 | low | low | high | high | high | low |
| Martindale 2018 | low | high | low | high | low | low |
| Patel 2020 | low | low | low | high | low | low |
| Piantino 2021 | low | low | low | low | low | low |
| Riedy 2016 | low | high | low | high | low | low |
| Tate 2017 | low | high | low | low | low | low |
| Wang 2022 | low | low | high | low | low | low |
| Donna-Ferreira 2019 | high | high | high | low | low | low |
| Liu 2016 | low | high | low | high | low | low |
| **Lead** | | | | | | |
| Bleecker 2007 | low | high | low | low | low | low |
| Schwartz 2010 | low | high | low | high | low | low |
| **Pesticides** | | | | | | |
| Hebert 2000 | low | low | high | low | low | low |
| **Contact sports** | | | | | | |
| Lee 2017 | low | low | low | low | low | high |
| Strain 2013 | high | high | low | low | low | low |
| Zivadinov 2018 | high | high | high | low | low | low |
| **Diving** | | | | | | |
| Hutzelmann 2000 | high | high | high | low | low | low |
| Palmer 1992 | low | high | low | high | low | low |
| Rinck 1991 | high | high | low | low | low | low |
| Sipinen 1999 | low | high | low | high | low | low |
| **Aeronautical/high altitude** | | | | | | |
| Alperin 2017 | low | high | low | high | low | low |
| Barisano 2022 | low | low | low | low | high | low |
| Fueredi 1991 | high | low | high | low | low | low |
| Hupfeld 2022 | low | low | low | low | low | low |
| Jersey 2013 | high | high | low | low | low | low |
| Kang 2014 | low | low | low | low | low | low |
| Lim 2012 | low | high | low | high | low | high |
| McGuire 2013 | low | high | high | high | low | low |
| McGuire 2014 | low | low | low | low | low | high |
| McGuire 2019 | low | low | low | low | high | low |
| Pimenta 1999 | high | high | high | high | low | high |
| **Toluene** | | | | | | |
| Aydin 2002 | high | high | high | high | low | low |
| Caldemeyer 1996 | low | high | low | high | low | low |
| Rosenberg 1988 | low | high | low | high | low | low |
| Rosenberg 1988 | high | high | low | high | low | low |
| **1,2-DCE** | | | | | | |
| Chen 2019 | low | high | high | high | low | low |
| Lai 2011 | low | high | high | high | low | low |
| Liu 2019 | high | high | high | high | low | low |
| Yang 2009 | low | high | high | high | low | low |
| **Carbon disulfide** | | | | | | |
| Cha 2002 | low | high | high | low | low | low |
| Cho 2002 | high | high | low | low | low | low |
| Huang 1996 | high | high | low | high | low | low |
| Nishiwaki 2004 | low | low | low | low | low | low |
| **Solvents** | | | | | | |
| DelBigio 2009 | low | high | low | low | low | low |
| Keski-Santti 2009 | low | low | low | low | low | low |
| Thuomas 1996 | low | high | high | high | low | low |
| Yamanouchi 1995 | high | high | low | high | high | low |

Notes on quality assessment: UC assessed quality using the Risk of Bias Assessment tool for Non-randomized Studies) Tool, with an additional co-author (YC) additionally independently assessing a random 33% sample. Examples of high risk of bias relating to the research question (available on request) were agreed between authors in advance to ensure that criteria (outlined in the main manuscript) were consistently applied.

## Supplementary Table 4. Summary of studies and sample sizes included in meta-analyses vs narrative synthesis according to substance/occupational exposure

| **Substance/occupation** | **Total no. included studies** | **No. studies in meta-analyses** | **No. studies in narrative review** |
| --- | --- | --- | --- |
| Carbon monoxide | 29 studies (n=1,409) | 28 studies  (n=1,373)  Control arm of case-control study excluded (n=16) | 1 study  (n=20) |
| Carbon disulphide | 4 studies  (n=797) | 3 studies  (n=131) | 1 study  (n=666) |
| Contact sports | 3 studies  (n=629) | 0 | 3 studies  (n=629) |
| 1,2-Dichloroethane | 4 studies  (n=40) | 4 studies  (n=40) | 0 |
| Diving | 4 studies  (n=266) | 4 studies  (n=172) | 0 |
| High altitude | 11 studies  (n=950) | 5 studies  (n=164)  Control arm of case-control study excluded (n=42) | 6 studies  (n=744) |
| Lead | 2 studies  (n=423) | 0 | 2 studies  (n=423) |
| Military | 10 studies  (n=18,893) | 0 | 10 studies;  (n=18,893) |
| Pesticides/fertilisers | 1 population  (n=8,623) | 0 | 1 study  (n=8623) |
| Miscellaneous solvents | 4 studies  (n=251) | 0 | 4 studies  (n=251) |
| Toluene | 4 studies  (n=64) | 4 studies  (n=64) | 0 |
| broad occupational categories in population-based dementia studies | 9 studies (n=15,118) | 0 | 9 studies (n=15,118) |

## Supplementary Table 5*.* Summary of case control studies

| **Author (year)** | **Substance** | **Key findings** |
| --- | --- | --- |
| Fueredi  (1991) | High altitude/aeronautical | The 19 subjects in the experimental group had a statistically higher number (p= .05) of white matter lesions (more than 152) than the control group (22 lesions), and 37% of the experimental group had more than 20 white matter lesions each (seven of 19 subjects) while only 18% of the control group had 10 or 11 lesions each. The experimental group had a five times higher risk than the control group of having highgrade lesions, and a high statistical correlation (p = .02) was found between the number and severity of lesions in the experimental group as compared with the control group when linear trend analysis was performed. The distribution of white matter lesions was 100% in the centrum semiovale and 50% in the optic radiations. Additionally, three subjects in the experimental group had two internal capsule lesions and one basal gangliar lesion. Statistical analysis of our data revealed that the exposed (experimental) group had a statistically significant increase in the number of PWMLs as compared with the control group (Kruskai-Wallis test, p = .05). An odds ratio was also calculated, which showed a fivefold increase in the likelihood of an exposed individual having a grade 3 or 4 lesion compared with an age-matched control subject. The 90% confidence interval for the odds ratio ranged from an increased risk of 1.13 times to 22.2 times. |
| Fukuhara  (1996) | Carbon Monoxide | 15 acute carbon monoxide poisoning cases and 16 age and sex matched controls. 7/15 had lesions in the globus pallidum bilaterally, 4/15 had lesions in the parietotemporooccipital lobe bilaterally, and 5/15 had multiple deep white matter lesions. No documentation of control group brain scan findings |
| Hutzelmann (2000) | Diving | Compressed air divers with uneventful diving history: 27 commercial and 32 navy divers without history of neurological decompression illness, 48 controls matched for age/BMI/alcohol/smoking. All cerebral lesions supratentorial except one cerebellar lesion, lesions were predominantly located in subcortical and central white matter of the parietal lobes and in the basal ganglia. No periventricular lesions were found. Nonfocal white matter hyperintense lesions were observed in 22/59 (37.3%) of divers and in 23/48 (47.9%) of controls, non=significant. 18% of nondivers had > 3 hyperintense spots in their white matter vs 12% of divers |
| Lee  (2017) | Contact sports | 499 cases vs 62 controls. Licensed professional fighters including active fighters (boxers, mixed martial artists) retired professional fighters, age-matched healthy controls. The prevalence of nonspecific WM changes was similar between groups. Fighters had a prevalence of cerebral microhemorrhage (4.2% vs0% for controls, P .152). WMCs were present in 118/499 (23.6%) fighters and 12/62 (19.4%) controls (P .525). CMHs were present in 21/498 (4.2%) fighters and 0/62 controls (0%, P= .152). 59 CMHs identified: n=1 had 14 CMHs, n=1 had 10, and the remaining 35 CMHs were found in 19 fighters (mean, 1.5 CMHs per fighter). Almost all CMHs located near the cortex, only a few in the deep white matter and periventricular margins. In posterior fossa, most cases of CMH were in the inferior cerebellum. There were relatively few CMHs in the supraorbital convexities, deep gray matter, or corpus callosum. Most WMCs were located near the cortex, and only 2 were located in the periventricular margins. Fighters with WMC trended toward having a higher average of total fights (60 fights versus 46 in those without WMCs, P .098; 56 versus 42 in those without CSP, P .054). Fighters with CMH had a higher average of total fights than those without CMH, not significant (85 vs 47, P .530) |
| Lim  (2012) | High altitude/aeronautical | 31 cases + 31 controls: Asymptomatic brain lesions in pilots: a comparative study with non-flying personnel using brain MRI. There was no statistical difference between the pilots and non-flying personnel for WMH findings (54.8%/ vs. 32.3%, p=0.073 ). Number of WMH pilots 5.2 ( 9.44) nonflying personnel 4.6 (10.15), p= 0.825. no. of WMH not associated with aircraft type,, total flight hours, altitude. Of the factors related to flight, only the flying altitude (OR 1.005, (1.001-1.009)]) was significantly related to the presence of WMH on multivariate analysis |
| McGuire (2013) | High altitude/aeronautical | 102 cases, 91, controls, U-2 pilot occupational exposure to hypobaria in active duty members in the US airforce / controls =active-duty military members with a doctorate degree assigned to duty. U-2 pilots demonstrated an increase in volume (394%; p = 0.004) and number (295%; p < 0.001) of WMH. Analysis of regional distribution demonstrated WMH more uniformly distributed throughout the brain in U-2 pilots compared with mainly frontal distribution in controls. Pilots with occupational exposure to hypobaria showed a significant increase in WMH lesion volume and number. Population-wide overlap of individual subcortical WMH  demonstrated both a much larger number of lesions in pilots and a more uniform regional distribution of WMH than in normal controls. Pilots demonstrated a nearly 4-fold increase in volume (375%) and a 3-fold increase in the number (294%) of WMH; this difference was significant for both the raw data and the site-specific adjusted data. Spearman correlation coefficient between lesion volume and age approached significance for the normal controls but not for the pilots (p 5 0.07 and 0.16; r 5 0.19 and 0.14 for controls and pilots, respectively). The correlation coefficients between the number of lesions and age were not different (p 5 0.14 and 0.2; r 5 0.15 and 0.13 for controls and pilots, respectively). Likewise, the correlation co efficients between the volume and number of WMH and the number of U-2 flight hours were not different for the pilots (p . 0.5; r 5 0.05 and 0.03 for volume and number, respectively).This study demonstrated that pilots exposed to hypobaria had increased volume and number of sub cortical WMH compared with a healthy, age- and education-matched normative population. |
| McGuire (2014) | High altitude/aeronautical | Eighty-three altitude chamber personnel, active duty members of the US military exposed to nonhypoxic hypobaria (PHY), 105 U-2 pilots (U2P), and 148 age- controlled and healthmatched doctorate degree controls (DOC). Subcortical WMHs were more prevalent in PHY (volume p 5 0.011/count p 5 0.019) and U2P (volume p < 0.001/count p < 0.001) when compared to DOC, whereas PHY were not significantly different than U2P. |
| McGuire (2019) | High altitude/aeronautical | N = 64 healthy aircrew undergoing standard US Air Force altitude chamber training and compared to N = 60 controls not exposed to hypobaria. No vascular RFs. No significant changes were observed in structural measurements including manually traced WMH/total WMH volumes. |
| Nishiwaki  (2004) | Carbon disulphide | A total of 666 subjects (217 exposed, 125 ex-exposed, and 324 referent subjects) who twice received brain MRI were subjected to analysis. Exposed subjects showed a significantly higher risk for an increase in the number of hyperintense spots (HIS) over six years. Odds ratios adjusted for possible confounders in the exposed and ex-exposed workers were 2.27 (95% CI 1.37 to 3.76) and 1.33 (95% CI 0.70 to 2.54), respectively. The odds ratio adjusted for age in logistic regression analysis, in which the dependent variable was ‘‘increased’’ or ‘‘unchanged’’ in number of HIS over six years, was 2.56 (95% CI 1.59 to 4.10) in the exposed group and 1.24 (95% CI  0.68 to 2.27) in the ex-exposed group. The multivariate adjusted odds ratio in the exposed group (adjusted OR 2.27,  95% CI 1.37 to 3.76) remained significantly higher compared to the referent group. Age was a significant risk factor for an  increase in number of HIS, separate from exposure to CS2. |
| Patel  (2020) | Military | 71 Cases=military personnel persistent symptoms related to active duty mild TBI secondary to blast/blunt force injuries vs 75 non-TBI healthy controls. The odds of >=1 WMH in the brain-injured group was about 3.5 times the odds for healthy controls (95% CI, 1.58-7.72; P = .002) after adjustment for age. Similar findings were seen for frontal lobe WMH (OR = 2.43; 95% CI, 1.16–5.08; P = .02). Median total WMHs in the mTBI and healthy controls groups were 3 (range, 0–108) and 1 (range, 0–83), respectively. Increasing white matter lesion burden in the mTBI-versus-healthy controls cohort was not observed in other regions |
| Riedy  (2016) | Military | 834 cases, 42 controls. The most common finding in our primarily mild chronic TBI population was the presence of dilated perivascular spaces (64.3% [536 of 834] vs controls 57.1% of n=42); OR, 1.35 [95% confidence interval: 0.72, 2.53], p=0.22) and T2-weighted hyperintense areas in the white matter (51.8% [432 of 834] vs controls 38.1% of n=16/42; OR, 1.75 [95% confidence interval: 0.92, 3.30], p=0.57). Microhemorrhage, 6.64 [95% confidence interval: 0.40, 109.22]) of TBI participants  were noted in 7.2% (60 of 834; OR, and showed increased incidence with TBI severity (P < .001, moderate and severe vs mild). T2-weighted hyperintense areas and microhemorrhages did not collocate by visual inspection. Postcontrast T2-weighted FLAIR enhancement observed in 13.7% (114 of 834 vs 2.4% of n=42; OR, 6.49 [95% confidence interval: 0.88, 47.66], p=0.018) of participants with TBI, and surface veins were the most likely source. The controls had no evidence of microhemorrhage. |
| Rinck (1991) | Diving | 70 cases and 47 controls: professional male divers with ≥1year of professional diving experience; controls policemen and offshore workers. Spots of high signal intensity in white matter on PD- and/or T2-weighted spin-echo images were detected in 42% of the control subjects and in 34% of the divers. In controls, prevalence of >3 changes was related to smoking, use of alcohol, head trauma, age>35 years, and multiple cerebrovascular risk factors. This relationship was not present in the divers. The prevalence of changes in divers was inversely related to diving depth, amount of diving, participation in "unsafe diving," and decompression sickness. |
| Strain (2013) | Contact sports | 10 cases, 20 controls. Former professional American football players with cognitive deficits vs healthy matched controls, showed that total and deep WMH volumes were higher in the player group (mean 8.13 mL vs 2.38mL) but periventricular WMH volumes were not significantly different between groups, mean age 61.8 years |
| Thuomas  (1996) | Solvents | WMH in 11/30 exposed and in 0/40 controls |
| Zivadinov  (2018) | Contact sports | Retired male professional contact sport (football and hockey) players 21 contact sport and 21 noncontact sport. No structural differences in presence, number, and volume of focal white matter signal abnormalities or in number, and volume of cerebral microbleeds. 12 (57.1%) of contact sport athletes and 11 (52.4%) of non-contact controls presented with White Matter-Structural Abnormalities (WM-SAs). There were no significant differences between contact sport athletes and non-contact controls for the total number and volume of WM-SAs. No significant differences were found for various CMBs outcomes between contact sport athletes and non-contact controls. However, more non-contact athlete controls (7, 33%), compared to contact sport athletes (2, 9.5%) presented with at least one CMB (p=.067), non-significant. CMB number (.6 vs .3, 95% CI [−.5 – .9], d=.20, p=. 542) and volume (11.2mm3 vs. 2.3mm3 , 95% CI [−.7 – 18.6], d=.58, p=.077) slightly higher in non-contact athletes vs contact sport athletes. |

## Supplementary Table 6*.* Summary of longitudinal study findings

(Full population longitudinal)

In summary, carbon monoxide studies assessing WMH reported persistence/no change at six months,^16^ a combination of WMH increase and decrease over one year,^25^ WMH persistence or worsening over 8-18 months.^18^ In one longitudinal carbon disulphide study, WMH increased over six years in factory workers exposed to viscose rayon but not in controls.^46^ Two astronaut studies found PVS volume increase in a six-month interval pre- and post-flight^68,69^ and one study assessing high-altitude exposure detected no significant changes in total WMH volume at 24 and 72 hours post-exposure.^61^ In former organolead workers undergoing two MRI scans with a 5-year interval did not detect worsening Cardiovascular Health Study WMH scores in cases vs controls. A final convenience sample from a military study found that blast exposure severity <9 years prior, but not blast number, was associated with change in WMH number and volume.^80^ Ten further studies were predominantly cross-sectional but a subset had longitudinal imaging. (References accessible in main paper).

| **Study**  **(Year)** | **Population** | **Study design** | ***n*** | **Mean age** | **MRI vs CT** | **Longitudinal change** | **Duration and concentration of exposure** | **Exposure-imaging interval** |
| --- | --- | --- | --- | --- | --- | --- | --- | --- |
| Barisano 2022 | astronauts | Longitudinal observational study | 37 | 48.1 | MRI | MRI performed within days of return detected increased volumes of post-flight PVS (pre-flight white matter PVS mean in NASA space station group 1152mm (SD 559) vs post-flight 1435mm (SD 698), p<0.001; significant findings also detected in basal ganglia PVS; no change detected in controls). | spaceflight lasting six months | brain MRI scans acquired before and within 2 wk (mean 4.8 [SD 2.2] days) after long-duration spaceflight on the ISS (∼180 d) |
| Hupfeld 2022 | astronauts | Longitudinal observational study | 26 | 47.46 | MRI | Following spaceflight, there was an increase in total PVS volume from pre- to post-flight in novice astronauts (estimate for change in total PVS volume (mm^3^/cm^3^ of WM) = 0.16; [SE 0.06]; p = 0.020) but change in PVS number. Experienced astronauts had decreased PVS volumes postflight. There was an additional trend towards higher pre-flight PVS load and greater flight experience (r=0.60–0.71; p > 0.05). | spaceflight lasting six months | Mean 4.53 (SD 1.13) days |
| Martindale 2018 | veterans with history of blast exposure in Iraq and Afghanistan without a lifetime history of TBI | convenience sample from longitudinal observational study | 19 | 39 | MRI | MRI WMH changes were associated with severity of blast exposure. higher quality of imaging at Time2 was an influence on WMH increase, as several of the ‘new’ hyperintense areas were faintly present on the Time1 images.  Regarding change between T1 and T2,there were significant associations between severity of blast exposure on changes in WMH number (r = .54, p = .018) and volume  (r = .50, p = .031). There was no association between number of blasts on change in WMH number (r = –.07, p = .769) or volume (r = –.09, p = .720), or new TBI and change in WMH number (r = .21, p = .384) or volume (r = .27, p = .257). | average 1-4 combat deployments | less than 9 years (2001-2010) |
| McGuire 2019 | N = 64 healthy aircrew undergoing standard US Air Force altitude chamber training and compared to N = 60 controls not exposed to hypobaria | Cross-sectional case-control study | 124 | 21 | MRI | No significant changes were observed in structural measurements including manually traced WMH/total WMH volume | Standard protocol at 5,000 ft (exposure 12.2 psi/632 mmHg/ambient air O2 via aviator mask>500 mmHg) followed by 30 min of denitrogenation on 100% O2 at sea level via an aviator mask. Trainees then ascend to 25,000 ft (exposure 5.45 psi/282 mmHg/ambient air O2 via aviator mask >200 mmHg) where they remain for 20 min. Trainees remove their aviator masks, redoning with the first onset of hypoxic symptoms. Total duration of mask removal at altitude is approximately 2–4 min with an O2 saturation reaching 65–75% (exposure 5.45 psi/282 mmHg/ambient air O2 59 mmHg). Following recovery on 100% O2 via aviator mask, trainees descend to 18,000 ft followed by descent to sea level | 24h and 72h post-exposure |
| Nishiwaki 2004 | viscose rayon factory workers across 11 factories in Japan and referent workers - (217 exposed, 125 ex-exposed, and 324 referent subjects) | Longitudinal case-control study | 666 | 35.9 | MRI longitudinal in 666 | Changes in the number of hyperintense spots in T2 weighted images (HIS), which point to so-called "silent cerebral infarctions", were evaluated over six years. Exposed subjects showed a significantly higher risk for an increase in the number of HIS over six years. Odds ratios adjusted for possible confounders in the exposed and ex-exposed workers were 2.27 (95% CI 1.37 to 3.76) and 1.33 (95% CI 0.70 to 2.54), respectively. The odds ratio adjusted for age in logistic regression analysis, in which the dependent variable was ‘‘increased’’ or ‘‘unchanged’’ in number of HIS over six years, was 2.56 (95% CI 1.59 to 4.10) in the exposed group and 1.24 (95% CI 0.68 to 2.27) in the ex-exposed group. The multivariate adjusted odds ratio in the exposed group (adjusted OR 2.27, 95% CI 1.37 to 3.76) remained significantly higher compared to the referent group. Age was a significant risk factor for an increase in number of HIS, separate from exposure to CS2. Of subjects who were globally assessed as ‘‘increased’’, 26 exposed (12.0%), 13 ex-exposed (10.4%), and 20 referent (6.2%) subjects showed at least one category increase. When this criterion was applied, the multivariate adjusted odds ratio showed marginally significant result in the exposed group (adjusted OR 1.89, 95% CI 0.97 to 3.68) compared to the referent group | The geometric mean CS2 (atmospheric, ppm) and TTCA (urinary, mg/g creatinine) concentrations for the past six years were 4.9 and 1.6 for all exposed workers, 5.8 and 1.9 for spinning/refining workers, and 2.7 and 0.9 for other exposed workers, respectively. Mean duration of exposure to the end of the study was 19.6 years for the exposed workers. | 251 remained to be exposed to CS2 until the end of the observation period (exposed workers), and 140 workers had their exposure truncated (ex-exposed workers). mean and median periods from the cessation of exposure to the follow up survey in the ex-exposed subjects were 4.0 and 4.1 years (range 0.8–6.9 years) |
| Parkinson 2002 | carbon monoxide poisoned patients with COHb level ≥10% referred from emergency department, consecutive, plus age and sex-matched normal control subjects selected from a normative database | longitudinal cohort study | 73 | 34.7 | MRI | The WMH in CO-poisoned patients did not change from day 1 to 6 months. Only WMH in the centrum semiovale were significantly associated with cognitive impairments. No significant differences were found for PVWMH or CSWMH between day 1 and 2 weeks, between day 1 and 6 months, or between 2 weeks and 6 months. There were no significant correlations between WMH and COHb level and duration of CO exposure at any of the three scan times. | Included CO-poisoned patients had a mean COHb level of 22.0 +/- 10.6% (range 1.2 to 39.0%), CO exposure duration (n 57) of 15.2 +/- 47.5 hours (range 0.25 to 308 hours. Mean CO exposure duration was 22 +/- 70 hours (range 15 minutes to 20 days). Causes of CO exposure included exposure to automobile exhaust (42%) (half [21%] were intentionally poisoned), internal combustion engine exhaust (29%), faulty furnaces (22%), and fumes from smoldering charcoal briquettes (6%) and from fire (1%) | MRI acquired within 24-36 hrs, repeated 2 weeks and 6-months |
| Pavese 1999 | Patients referred to hyperbaric oxygen therapy specialist centre for CO intoxication: 22 with acute exposure (domestic heating system failures), 8 chronic exposure due to domestic heating system failures x 22 days | Longitudinal observational study | 22 | 39 | MRI | - 11/22 had WMH in the acute CO exposure group: in n=3, one-year MRI showed marked decrease in extent and signal intensity of WMH especially in the  frontal and parietal centrum semiovale.  n=1 had a worsening of MRI white matter abnormalities at month two but subsequent MRI showed decreasing WMH. n=1 MRI examination (month 3) showed increasing WMH and new bilateral thalamic lesions. In n=3, one-year MRI showed no changes in 3 cases. n=1, decrease in centrum semiovale WMH n=2 lost to follow-up  8/22 had WMH in chronic CO exopsure group: In n=1, widespread WMH unchanged at 1-year follow-up. The other 7 patients in this group showed no baseline MRI abnormalities, not reported what their followup MRI showed. | acute mean 1.4 hrs vs 5 hrs and chronic mean 22 days (range 12-30 days) | one month (first scan) and 1 year (follow-up scan) |
| Schwartz 2010 | former organolead workers from a chemical manufacturing plant , involved in the manufacture of tetraethyl lead + tetramethyl lead | Longitudinal  observational study | 362 | 60.8 | MRI | For the change in CHS white matter lesion grade score (CHS2 minus CHS1), 6 persons (1.7%) improved by one category, 87 persons (24.0%) were unchanged, 134 persons (36.9%) worsened one category, 100 persons (27.6%) worsened by two categories, 24 persons (6.6%) by three categories, and 12 (3.3%) by four or five categories. Neither peak tibia lead nor control status were associated with change in CHS sores. Baseline age and increasing duration between MRIs were associated with increases in CHS scores (beta 0.055, P 0.001 and beta 0.286, P 0.05, respectively). | mean 8.5 (9.6) years of employment in lead factory | mean 18.0 (10.9) years since last lead exposure at study recruitment (which was approx four years before first MRI) |
| Vieregge 1989 | Patients with coma and midbrain syndrome due to acute CO poisoning given normobaric oxygen therapy. Diagnosis of CO poisoning proved either circumstantially or by COHb determination. Exposure to defective heating equipment in n=4, n=1 inhaled automobile exhaust fumes in a suicide attempt. | Longitudinal observational study | 4 | 35.5 | CT/MRI (MRI in n=3) | n=4. Individual findings for each case: Patient l. a Axial CT 4 days after CO poisoning: bi lateral low-density areas in the globus pallidus, b CT scan 24 days after CO poisoning: slight enlargement of ventricles and cortical sulci.  Low-density areas in the globus pallidus no longer visible, c-e MRI 18 months after CO poisoning: (c) show a small, hypointense lesion in the globus pallidus (d) with small bilateral hyperintense pallidal lesions corresponding to cicatricial cysts. Here and in the neighbouring section (e) a dot-like hyperintense lesion in the frontal white matter is seen.  Patient 2. No data reported so not included  Patient 3. a Axial CT about 10 h after CO poisoning: bilateral low-density areas in the globus pallidus, b CT scan 8 months after CO poisoning: bilateral low-density areas of the globus pallidus are still present with slight enlargement of ventricles and cortical sulci. a, b A small cyst-like lesion is also seen in the right anterior limb of the internal capsule adjacent to the head of the caudate nucleus. c MRI 18 months after CO poisoning: unilateral hyperintense lesion between the left internal capsule and globus pallidus.  Patient 4. A Axial CT scan about 8 h after CO poisoning: bilateral low-density areas in the globus pallidus, b CT 24 days after CO poisoning: no pallidal lesions visible  Patient 5. a Axial CT scan 14 days after CO poisoning: bilateral low-density areas in the globus pallidus, b CT 24 days after CO poisoning: conspicuous low-density changes especially in the subcortical white matter. | CO levels 22, 25,25, 0 (n=4) | CT or MRI within hours-days; for n=3 with follow up MRI/CT< occurred from 8-18months |

## Supplementary Figure 1. Meta-regression of SVD prevalence in carbon monoxide studies adjusting for age


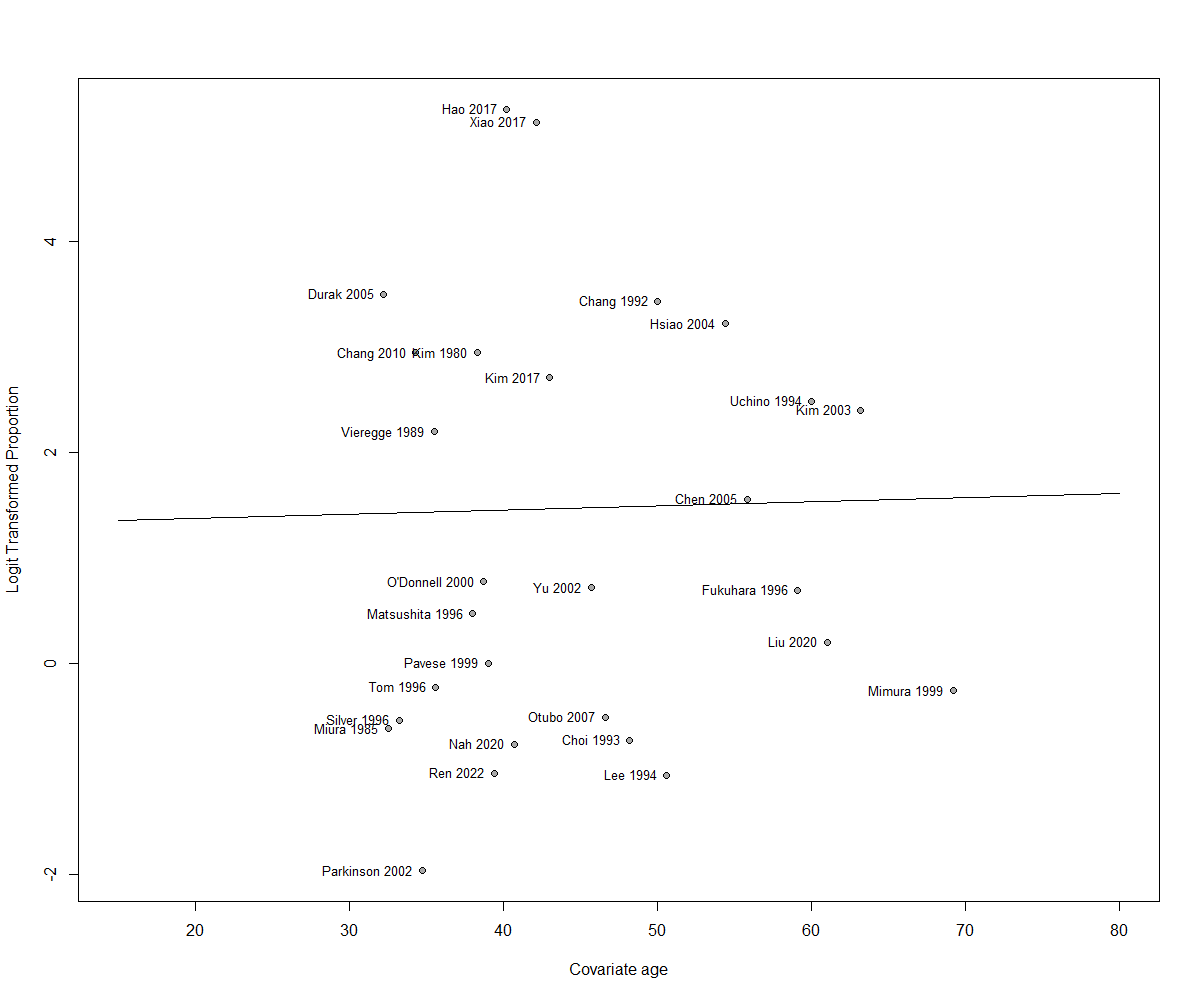


## Supplementary Table 7. Typical exposure settings for each of the substances studied

Source: Centers for Disease Control and Prevention

| **Substance** | **Settings where commonly found** |
| --- | --- |
| Carbon monoxide | Incomplete burning of carbon-based fuels, e.g. gas, oil, wood and coal; faulty heating systems, mining, chemical manufacturing, food packaging; urban pollution, vehicle exhausts, power washers, fire places, charcoal grills, marine engines, forklifts, portable generators |
| Carbon disulphide | Factory rubber processing, cellophane production, rayon fabric, carbon tetrachloride production, perfumes, varnish, solvents, insecticides |
| 1,2-dichloroethane | PVC pipes, furniture and automobile upholstery, wall coverings, varnishing, housewares, car parts, textile cleaning/drycleaning, metal degreasing, grain fumigation, chemical industry, printing and publishing, electricals, trade, leather, machinery |
| Toluene | Paint and lacquer thinner, dyes, nail salons, rubber cleaning and drying agent, timber, underground fuel storage, motor and aviation fuels and chemical industries, glues, adhesives, inks, cleaning liquids |
| Miscellaneous solvents | Paints, varnishes, lacquers, adhesives, glues, coatings, degreasing agents, cleaning products, plastics, textiles, agricultural products, polymers, pharmaceuticals, dyes, printing inks |
| Lead | Lead-based paint/dust including refurbishment of old houses, water pipes, construction, contaminated soil, leaded gasoline, battery recycling, shipbuilding, roadwork, finishing furniture, ceramic glazing, underground storage tank leakage, lead ore mining, smelting, waste incinerators |
